# Bone tools, carnivore chewing and heavy percussion: assessing conflicting interpretations of Lower and Upper Palaeolithic bone assemblages

**DOI:** 10.1098/rsos.231163

**Published:** 2024-01-03

**Authors:** Simon A. Parfitt, Silvia M. Bello

**Affiliations:** ^1^ Institute of Archaeology, University College London, 31–34 Gordon Square, London WC1H 0PY, UK; ^2^ Centre of Human Evolution Research, Natural History Museum, Cromwell Road, London SW7 5BD, UK

**Keywords:** evolution of technology, bone tools, taphonomy, Palaeolithic archaeology, Acheulean, Magdalenian

## Abstract

The use of bone tools by early humans has provided valuable insights into their technology, behaviour and cognitive abilities. However, identifying minimally modified or unshaped Palaeolithic osseous tools can be challenging, particularly when they are mixed with bones altered by natural taphonomic processes. This has hampered the study of key technical innovations, such as the use of bones, antlers and teeth as hammers or pressure-flakers to work (knap) stone tools. Bones chewed by carnivores can resemble osseous knapping tools and have sometimes been mistaken for them. In this paper, we review recent advances in the study of osseous knapping tools with a focus on two Palaeolithic sites in the UK, the Acheulean Horse Butchery Site at Boxgrove and the Magdalenian site of Gough's Cave, where knapping tools were mis-attributed to carnivore chewing. These osseous knapping tools are investigated using microscopy, high-resolution imaging and comparisons with experimental knapping tools. This allows for new insights into human behaviour at these sites and opens fresh avenues for future research.

## 1. Introduction

The origins of human technology can be traced back to the Late Pliocene and Early Pleistocene in eastern Africa, as evidenced by archaeological discoveries of stone tools from sites dating to perhaps as early as 3.3 Myr [1,2]. These early stone tools were simple but effective, consisting of sharp-edged cutting and scraping tools and hammerstones that allowed early humans to access novel food resources and process them in ways not available to other contemporary primates [1,3,4]. One of the most important but least understood components of this early toolkit is the percussors, which were used to detach flakes from the core and to shape and resharpen the cutting edges. The relatively small number of known knapping tools from the early stages of the Palaeolithic can be attributed, in part, to the difficulty involved in distinguishing pebbles and osseous tools used as hammers or pressure-flakers for stone-working tasks and distinguishing them from tools used for other pounding activities [5–7]. Additionally, bones that have been broken with a stone hammer to extract marrow [8] or bones modified by natural processes, such as carnivore chewing [9–12], further complicate the identification process.

The earliest stages of stone working employed ‘hard’ hammers, usually stone cobbles, to detach flakes from cores and work the flake edges to create simple scraping or cutting tools [13]. However, as stone tool production became more refined, ‘soft’ hammers of bone, antler or wood were adopted to produce symmetrical and thinner standardized lithic tools. The development of these soft hammers was a critical technological innovation that allowed early humans to produce more complex and refined lithic tools [13,14]. By studying these knapping tools and their use, it is possible to gain valuable insights into the cognitive and technological abilities of early humans and the evolution of tool-making techniques over time, as well as social interactions and knowledge transfer between different human groups.

Despite a rich research tradition dating back to the late nineteenth century in Europe, the identification and interpretation of osseous knapping tools remain contentious [15]. This challenge arises from the difficulty of differentiating them from pseudotools (i.e. naturally modified bones resembling artefacts) that result from natural modifications. For example, mammalian gnawing (Binford [8] versus Chase [16]), crocodile feeding (Backwell & d'Errico [9,10] versus Pante *et al*. [12]) and natural abrasions [17] can modify bones in ways that resemble knapping damage. Additionally, impact damage resulting from activities such as marrow and bone grease processing and various types of pounding activities involving osseous hammers or anvils can also create bone surface modifications that can be difficult to distinguish from knapping marks [18–20].

In this paper, we provide a brief overview of recent developments in the study of Palaeolithic osseous knapping tool industries. We then discuss whether the apparent rarity and variations in the types of knapping tools observed in Palaeolithic assemblages reflect early hominin behaviour or result from analytical biases during the excavation or in the laboratory. The challenges of identifying osseous knapping tools are examined, with reference to two case studies where expert zooarchaeologists have come to different interpretations of Palaeolithic knapping tools. These examples are taken from the early Middle Pleistocene Horse Butchery Site (HBS) at Boxgrove, and the Magdalenian occupation at Gough's Cave, both in the UK. We argue that these case studies highlight the difficulties inherent in identifying osseous knapping tools, particularly when they are fragmentary or show ambiguous modification patterns. Furthermore, we suggest that a more comprehensive and rigorous approach to the analysis of osseous tools is needed to overcome these challenges to better understand the behaviour and activities of early humans.

## 2. Review of Palaeolithic knapping tools

The earliest knapping tools using bone date to the Lower Palaeolithic [15], with the simplest types being unworked long-bone shaft fragments. This type of knapping tool is particularly prevalent in European Middle Palaeolithic sites, where the function of these pitted bones (compresseurs, percuteurs, retouchoirs) was first recognized during the late nineteenth and early twentieth centuries [21–24].

Other types of osseous knapping tools appear during the Lower Palaeolithic, including anvils used in bipolar (hammer-and-anvil) knapping [19] and worked antler hammers, the earliest of which are know from a late Acheulean context. Later innovations include the use of a varied range of punches and pressure-flaking tools that are associated with the Upper Palaeolithic. Canines of large carnivores, such as lions, were also used as knapping tools in the Aurignacian in western Europe, dating to around 40 000–28 000 years ago (e.g. [25]). The use of carnivore canines as knapping tools is a characteristic feature of the Aurignacian lithic technology and is thought to have had symbolic significance, perhaps as a marker of prestige or a cultural tradition.

The study of used bones, as opposed to regularly fashioned Palaeolithic bone tools, has received renewed attention in the last two decades [20,26], [27, fig. 7]. This recent surge in interest in unshaped bone tools has its origins in the 1980s, in part prompted by influential publications by Brain [28], Binford [8] and Shipman [29], who laid the experimental groundwork for the discipline of vertebrate taphonomy and its application to archaeological problems.

One important outcome of these studies was the recognition that several purported early bone tool industries were entirely natural, the result of accumulation and feeding on ungulate carcasses and bones by large carnivores, such as hyaenas, leopards and wolves [8,28]. This helped to refute many examples of early bone tools that had previously been attributed to human modification. Binford [8] also argued that human modification of bones could be distinguished from non-human biotic and abiotic agencies using a pattern recognition approach, without resorting to the microscopic methods that were being developed by Shipman [30] at that time. The use of microscopy has since been widely adopted by researchers studying Palaeolithic bone tools.

Recent fieldwork and discoveries of osseous knapping tools in museum collections have shed new light on the origin, types and spread of this technology during human evolution. While the most extensively examined record is from Europe, new discoveries from regions beyond this ‘core’ area have posed challenges to pre-existing notions of the development of osseous knapping tool technologies. Additionally, these discoveries have underscored the necessity of sustained research, particularly in regions where taphonomic studies of bone assemblages are in their early stages [31].

### 2.1. Lower Palaeolithic origins

The earliest known assemblage of bones and antlers used to knap lithic tools is dated to *ca* 500 000 years ago at Boxgrove, UK. These findings have been reported by Roberts & Parfitt [32], Smith [33], Stout *et al*. [34] and Pope *et al*. [35]. One of the most significant discoveries at Boxgrove is the assemblage of bone and antler hammers found alongside finely flaked Acheulean handaxes and rarer flake tools. The assemblage includes the largest collection of hard and soft knapping tools from an Acheulean context. Of particular note are the exceptional examples of knapping hammers made from antlers. Until discoveries at Boxgrove, the earliest known antler hammers were those used by Aurignacian knappers between 43 000 and 32 000 years ago at Geißenklösterle in Germany [36,37]. The use of antler as a raw material for making hammers is a significant technical advancement, indicating a high degree of technological investment. The antlers used at Boxgrove were methodically reduced to create usable hammers from the antlers of giant deer and red deer, demonstrating the deliberate selection of a raw material that is both tougher and more flexible than bone, making it an ideal material for knapping tools. These factors indicate a significant investment in time to procure suitable antlers and the skill to work them efficiently, resulting in the creation of highly effective tools that were curated as part of the portable tool kit used to shape and resharpen many handaxes.

In addition to the antler hammers, the Boxgrove assemblage also includes approximately 60 bones used as knapping percussors. Some of these bones were scraped to remove soft tissue (particularly periosteum) to expose the harder bone surface beneath, whereas others show deliberate shaping by percussions to improve the ergonomic properties of the hammers [38]. This assemblage is significant because some of these bone percussors were also curated implements, forming a transportable knapping kit that included flint hard hammers, antler hammers and handaxes. This kit included knapping tools that were carried from location to location and used over a relatively extended period to rejuvenate cutting tools when needed.

It is noteworthy that some of these curated implements occur in the same contexts as ad hoc bone-knapping tools that were used as the knappers found them to satisfy immediate needs, such as at a butchery site where resharpening of cutting tools was undertaken [38]. This demonstrates the behavioural flexibility of the early Middle Pleistocene hominin population at Boxgrove and their ability to deal with immediate needs and plan for future events.

Recent discoveries in museum collections have revealed previously unrecognized flint-knapping tools at the Clactonian-type site of Clacton-on-Sea (UK). These finds are particularly significant as it was previously believed that this relatively simple lithic technology, which dates to *ca* 400 000 years ago, was an entirely ‘hard’ hammer industry focused on the production of flakes that were subsequently modified to create unstandardized cutting and scraping implements [39]. At Clacton, bone percussors were likely used for the final flaking or resharpening of Clactonian scrapers and other flake tools [40]. The discovery of these hitherto unrecognized bone tools suggests that the Clactonian technology was more complex and used a wider range of materials and techniques than had previously been suggested.

Another significant advance in the study of Palaeolithic osseous tools arises from recent analyses of the faunal assemblage from the spear and horse butchery site (Schöningen 13 II-4) at Schöningen in Germany [19,41]. These include the earliest multipurpose bone tools represented by horse metapodials used as ‘hammers’ to crack marrow bones and knap flint, osseous anvils used in bipolar-knapping, and numerous retouchers [19]. The tools date to between about 337 000 and 300 000 years ago and were found alongside simply worked flint cutting and scraping tools that are distributed along a relatively narrow zone of the lakeshore. The abundance of bone-knapping tools at Schöningen can be explained by the absence of a nearby source of cobbles and pebbles, which necessitated the use of bones as hammers to knap flint and crack bones for marrow [20]. The bone tool assemblage is dominated by typical retouchers made of long-bone shaft fragments, but a range of other bone types were used as knapping tools, including ribs and more-or-less complete limb bones. Many of the bones used as hammers at Schöningen appear to derive from the butchery of ‘fresh’ horse carcasses, but other examples were selected from bones that had been lying around on the land surface for some time. Prior to use, preparatory scraping of both fresh and ‘old’ bones was undertaken to remove any remaining organic tissues or dirt from the area of the bone selected as the knapping surface [19]. Although dominantly derived from horses (the principal butchered animal), retouchers were also identified on the bones of deer and large bovids, and an exceptional example used the humerus of a sabre-toothed cat (*Homotherium latidens*)—one of the rarest of the large carnivores at the site [42].

A recent study conducted on flint chips that were found alongside three bone retouchers and the remains of a straight-tusked elephant from a lower horizon at Schöningen [43] has identified debris from the resharpening of flint tools. These resharpening ‘by-products’ indicate that tool working was undertaken on the spot. Moreover, the microwear traces and residues on the lithic artefacts suggest that they were used for tasks such as cutting wood, which were not directly associated with the processing of fresh animal tissues. These new insights challenge previous assumptions about the nature and purpose of the lithic technology at the Schöningen HBS, which was predominantly viewed as being geared towards the processing of animal carcasses. Instead, the findings support earlier indications that the inhabitants of this site were engaged in a variety of activities, some of which were not directly related to defleshing carcasses [44,45].

In southern Europe, somewhat earlier sites with very different suites of bone tools are exemplified by the 400 000-year-old Castel di Guido assemblage (Latium, central Italy), where a variety of bone tools, mostly made on elephant bone diaphysis encompassing bifaces, partial bifaces, unifaces, intermediate tools (wedges) and smoothers, but apparently lacking bones used as tools for working lithic artefacts [46].

### 2.2. Final Acheulean and early Middle Palaeolithic in Europe

Despite yielding fewer knapping tools, several other European later Middle Pleistocene sites have also yielded significant findings. These include sites with osseous knapping tools associated with final Acheulean and ‘transitional’ Middle Palaeolithic lithic industries, the latter including early prepared core (Levallosian) technologies. Well-studied examples include Atapuerca (Gran Dolina, TD10), Bolomor Cave and Cueva del Angel in Spain [47–50], as well as Cagny-l'Epinette, Orgnac 3, and Terra Amata in France [50–55]. The osseous knapping tools from these sites predominantly consist of long-bone shaft fragments.

### 2.3. Middle Palaeolithic in the European ‘core’ area

Retouchers are particularly numerous in European Mousterian contexts (e.g. [48,56–60]). As an example, the bone assemblage from the Quina Mousterian levels within the collapsed rock shelter at Chez-Pinaud (Charente-Maratime, France) provides a useful illustration of the characteristics of Mousterian knapping tools [61].

The Quina Mousterian levels at Chez-Pinaud, dating to MIS 4 (approx. 71 000–59 000 years ago), provide evidence of recurrent visits by highly mobile Neanderthal hunter–gatherers during the autumn and winter seasons. These visits were primarily focused on butchering hunted reindeer at the site. In addition to the 510 retouchers previously studied from earlier excavations, Baumann *et al*. [61] undertook a comprehensive analysis of an additional 83 retouchers, primarily consisting of diaphyseal splinters, from a recent excavation of the Quina levels. Baumann observed that bone retouchers were made equally from medium- and large-sized ungulates, suggesting that Neanderthals at the site deliberately selected the more robust bones from bone waste largely dominated by fragmentary bones of smaller-sized ungulates.

The Chez-Pinaud study is highly significant as it contributes to the recognition and growing evidence of a Neanderthal bone industry. This industry encompasses not only retouchers, but also a diverse assemblage of other bone tools, such as bevelled tools, retouched bones and a smooth-ended rib, which appear to have functioned as wedges/chisels, lateral cutting edges and a possible pressure flaker. It appears that these additional bone tool types were not previously recognized in earlier studies of the Chez-Pinaud Mousterian bone assemblages. In total, 103 bone tools were identified among the 3220 faunal remains, which is as many as the number of flint tools found, highlighting the significance of an ‘under recorded’ bone tool component in the Mousterian tool kit (see comments on Chagyrskaya below). The diversity of Mousterian bone tools, several of which have received only passing comment in earlier studies, is discussed by Gaudzinski [62], Leder *et al*. [63], Martisius *et al*. [64], Soressi *et al*. [65] and Majkić *et al*. [66].

### 2.4. Eastern Mediterranean region to East Asia

Archaeological research conducted beyond the European ‘core’ area of retoucher research has revealed the presence of retouchers at only two sites in the Levant (Qesem Cave, late Lower Palaeolithic [48,49] and Manot Cave, early Upper Palaeolithic [67]); a few sites in Siberia (e.g. Chagyrskaya Cave [68] and Denisova Cave [69]) and even as far east as central China (Lingjing [70]). These sites are situated in regions where different hominin groups were seemingly interacting in complex ways, and the presence of more than one contemporary hominin species in these regions further complicates the task of attributing these osseous knapping tools to specific hominin groups. As a result, understanding the associated behavioural repertoires has become a challenge for archaeological investigation.

Yeshurun *et al*. [67] note that virtually no retouchers have been recognized in the Levant, which is puzzling given the presence of numerous deeply stratified Middle-to-Upper Palaeolithic sites with rich and well-preserved large mammal assemblages that have been extensively studied by zooarchaeologists. They propose two hypotheses to explain the ‘non-identification’ of retouchers: either Levantine hominins did not habitually use bone retouchers or researchers working in the Levant have not yet identified them as such [67, p. 293]. These hypotheses are currently being tested by new research designs explicitly incorporating the search for retouching traces on bones to clarify this issue.

A significant advance in the study of early bone technology was made by Baumann *et al*.'s [68] investigation of the 50 000-year-old faunal remains from Chagyrskaya Cave (Altai, Siberia, Russia). The impetus of this research was inspired by Baumann's previous work on the Solutrean [71], which highlighted the potential of under-recorded earlier bone industries due to a lack of suitable methods and conceptual frameworks of study. Specifically, Baumann's study of the Chagyrskaya faunal collection aimed to ascertain whether a substantial bone industry existed at Neanderthal sites before the spread of anatomically modern humans in northern Eurasia. This study identified the most abundant and diverse assemblage of bone tools from a Neanderthal context [68] from deposits dating to approximately 50 000 years BP (late MIS 4 to early MIS 3), with a local (Micoquian) facies of a Mousterian lithic industry and several Neandertal remains. Their research recognized a substantial bone tool component, representing a systematic and organized production of a diverse range of bone tools, most of which were only marginally shaped, mostly by percussion. A traceological study of a subset of 780 examples revealed that retouchers comprised the majority (87.2%), alongside various intermediate tools, retouched pieces and tools with rounded tips.

The Chagyrskaya bone tool assemblage with its relatively diverse range of Neanderthal bone tools is comparable in some ways to the Neanderthal bone industry from Chez-Pinaud also studied by Baumann *et al*. [61]. What stands out in this comparison is the strikingly similar range and proportions of bone tools, with abundant retouchers comprising 88% at Chez-Pinaud and 94% at Chagyrskaya, followed by tools with lateral retouch (6.3% and 9.7%), bevelled tools (4.7% and 6.8%) and smooth-ended tools (1.8% and 2%), with multi-use tools accounting for 8.7% and 6.8%, respectively [61, p. 28]. This technology is attributed to a distinct Neanderthal bone industry primarily based on percussion, with limited use of scraping and abrasion techniques. These tools were employed in various tasks, including stone tool production, chiselling, polishing and cutting, not all of which were related to the butchery of carcasses.

Recent excavations at Denisova Cave have shed light on the diverse industry of unshaped and ‘formal’ (shaped) bone tools from the Middle Palaeolithic and Initial Upper Palaeolithic layers (45–48 ka) in the cave [69]. These tools were found in layers 11.4–11.2 in the East Chamber and include awls, intermediate tools and knives used to process organic materials such as leather and plant materials, as well as retouchers that were used to work lithic tools. Despite significant changes in stone tool industries from the Middle-to-Upper Palaeolithic, retouchers remained a constant component of the artefact assemblage. The excavations also allow quantification of these tools. In the East Chamber, for example, only eight retouchers (made from short lengths of cortical bone) were identified in a sample of approximately 10 000 bones analysed from layers 11.4–11.2 (Middle Palaeolithic to Initial Upper Palaeolithic), whereas a further six examples were found in the underlying Middle Palaeolithic layer 12. Retouchers from the lower horizons are primarily associated with Neanderthals based on sedaDNA, whereas sedaDNA from layer 11.2 includes a mix of Neanderthal, Denisovan and ancient modern human DNA [72]. The unworked bone tools in the upper levels of the site are found together with a variety of formal bone tools, including finely worked needles, pendants and personal ornaments crafted using more complex methods on a range of raw materials, such as ivory and ostrich eggshell [73]. However, it is currently not possible to link any of the hominin groups identified from the DNA evidence to the use of specific bone tool types from the upper deposits in this series.

Although the record of osseous knapping implements in East Asia is sparse, recent archaeological investigations at the Lingjing (Xuchang) site in Henan Province, China have shed new light on the subject. Doyon *et al*. [70] examined a sample of 277 bones from the assemblage of greater than 50 000 specimens and identified six limb-bone fragments and one antler of an axis deer bearing evidence for having been used as knapping tools. These bone tools were likely used as expedient retouchers to modify stone edges. In addition to these typical retouchers, the study identified evidence of percussion damage on weathered bones that was aimed at obtaining elongated splinters which were subsequently used as retouchers. The Lingjing site, dating to the Last Interglacial period (125–105 ka), is notable for its finds of two enigmatic hominin crania with morphological affinities to the Maba cranium from southern China and the Narmada neuro-cranium from India [74]. These hominin fossils raise questions about the presence and dispersal of early hominins in East Asia and their potential role in the development of bone tool technology in the region.

### 2.5. Africa

The paper by Turner *et al*. [75] highlights the rarity of evidence for bone osseous retouchers from the African continent. However, Turner's analysis of the faunal remains from the cave of Grotte des Pigeons at Taforalt (northeast Morocco) has identified 20 bone retouchers, which is the largest single collection of osseous knapping tools currently known from Africa. These retouchers come from levels spanning *ca* 85–24 ka cal BP, along with Middle Stone Age (MSA) Aterian to late MSA lithics. MSA retouchers have also been found at a few other sites in Africa, including Sidubu Cave and Blombos Cave in South Africa, and El Harhoura 2 and Contrebandiers Cave in Morocco [75–78].

### 2.6. Middle Palaeolithic to Upper Palaeolithic

In Europe, research by Toniato *et al*. [25] has revealed transitions and trends in the use of osseous retouchers by both Neanderthals and modern humans. This research has focused on sites in the Swabian Jura region of Germany, which record significant shifts in lithic technologies and knapping methods occurring throughout western Europe at this time. One of the most significant transitions observed is the replacement of Middle Palaeolithic technologies by Upper Palaeolithic technologies. During the Middle Palaeolithic period, Neanderthals appear to have used retouchers made exclusively from randomly collected bone fragments left over after butchering large mammal carcasses. These knapping implements were used to retouch stone tools and then discarded on the spot. Unworked (ad hoc) retouchers persist throughout the Upper Palaeolithic [79]. The orientation of the knapping marks suggests a change in the way the tools were used, as Middle Palaeolithic retouchers predominantly exhibit marks with a transverse orientation, whereas Aurignacian examples usually display knapping marks aligned parallel to the long axis of the retoucher. In addition to these unworked tools, the Aurignacian and later Upper Palaeolithic and Mesolithic technologies witnessed an increase in the diversity of knapping tools used, such as carnivore canines, modified tusks and antler bases and tines [36,80–82]. These tools served in different ways as percussors, pressure-flakers and anvils [25].

Although the Swabian Jura sites provide a less clear picture of the types of retouchers associated with Gravettian and Magdalenian industries, Taute [83] identified a decline in the use of osseous retouchers during the Gravettian and Magdalenian. The reasons for this apparent change in the use of knapping percussors from predominantly soft hammers to harder hammers are currently unclear. However, recent research has shown that unshaped ‘ad hoc’ retouchers may have been missed in previous studies of some later Upper Palaeolithic contexts, as shown by the recent discoveries of such tools in the Magdalenian faunal assemblage from Gough's Cave [84]. Similarly, earlier Solutrean assemblages also contain a significant proportion of unshaped knapping tools, including pressure-flakers used for manufacturing thin leaf-shaped bifaces [85,86]. Therefore, it is possible that the record of osseous retouchers is biased by the under-recording of ‘ad hoc’ retouchers that have yet to be recognized in other later Upper Palaeolithic contexts.

## 3. Organic knapping tools—a biased record?

Given the early recognition of retouchers as a distinct bone tool type and their widespread association with Palaeolithic stone industries from at least the early Middle Pleistocene to the end of the Ice Age, it is useful to understand why organic knapping implements have been historically overlooked, ignored or misidentified until recent decades.

### 3.1. Search images and analytical biases

A fundamental prerequisite for identifying retouchers in archaeological bone assemblages is linked to the analyst having an adequate search image for the types of modifications that are indicative for osseous knapping tools. This can come from personal experiences with knapping lithic tools, but perhaps more often the search image is limited to information gleaned from published illustrations and descriptions. Although there are widely consulted books on zooarchaeological taphonomy, such as Shipman [29], Brain [28], Lyman [87] and Fernández-Jalvo & Andrews [88], only Lyman mentions osseous knapping tools in passing.

One of the most influential of these seminal taphonomic studies is Binford's [8]. Binford's [8, pp. 44–46] contribution to the debate on bone retouchers was to dismiss Palaeolithic examples as pseudotools. He used modern animal-produced ‘compressor’ pitting and scoring on compact bones to illustrate his point [8, figs. 3.03–3.05]. Binford argued that ‘retouchers’ and ‘compressors’ from Palaeolithic sites (such as those illustrated by Henri-Martin [22–24], Movius [89], Bordes [90], Semenov [91], De Lumley [92]) were naturally created by carnivore chewing. Binford then applied his search image to a sample of Mousterian retouchers (*n* = ∼134) from Combe Grenal (France), which he interpreted as pseudotools showing pitting and scoring from carnivore teeth. Binford illustrated an example from Combe Grenal [8, fig. 4.41] and alludes to other examples (figured by Henri-Martin [23]), which showed striations produced during the removal of periosteum with overprinting by marks that he identified as pitting produced by carnivore teeth. These examples are particularly informative as they illustrate Binford's concept of pseudotools as applied to retouchers.

A parallel approach has been adopted by prehistorians, who applied a purely typological approach to the study of bone implements. Particularly relevant are publications by French prehistorians and the work of the Commission de la nomenclature sur l'industrie de l'os préhistorique. In 1974, H. Camps-Fabrer organized the first ‘International Meeting on Bone Industry’, which took place in Senanque (Vaucluse, France). At this meeting, the Commission developed a methodological approach to the typological characterization of Palaeolithic osseous artefacts. Until then, organic tools were exclusively recognized by comparisons with ethnographic evidence and their attribution to different typologies based on minimal recognizable features. With the development of this new approach in the 1960s and 1970s, the need to standardizes the methods of analysis became apparent, and in 1976, after the second ‘International Meeting on Bone Industry’, the Commission published a series of *Fiches typologiques* (typological sheets) relating to osseous implements and use wear [93]. *Cahier X. Compresseurs, Percuteurs, Retouchoirs*, edited by Maylène Patou-Mathis [26] is entirely dedicated to the recognition of knapping tools on antler, bone and teeth. Although this book aims to provide a comprehensive coverage of relevant modification types of knapping tools, it nevertheless bypasses the critical question of distinguishing natural modifications, such as those caused by carnivore chewing, from knapping modifications.

The divergence of approaches between taphonomists and prehistorians may have contributed to a lack of shared focus on identifying and differentiating between natural modifications and anthropogenic modifications related to the production and utilization of organic knapping tools. Reliance on published sources alone can lead to biased interpretations, as it is not always possible to capture the full range of bone modifications through illustrations and descriptions. Additionally, other biases and preconceptions of the analyst can affect the search image, leading to the under-recognition or misidentification of bone modifications in archaeological assemblages. To overcome these challenges, it is important to have a diverse range of analysts with different backgrounds and experiences, as well as incorporating blind testing and inter-observer reliability assessments in analyses to minimize individual biases. Furthermore, it is essential to continuously update and refine search images based on new discoveries and information.

### 3.2. Excavation and curatorial biases

There are several other reasons why the study of organic knapping tools made of materials such as wood, bone, antler and ivory has received so little attention until recently. Foremost is the fragility and perishability of organic knapping tools, which makes them less likely to survive in the archaeological record, especially when compared with those made of more durable materials, such as stone. Osseous knapping tools have also been overlooked due to the traditional emphasis on the study of formal and visually more impressive and elaborate osseous artefacts to the exclusion of ‘mundane’ unworked types (e.g. [84]).

Other significant factors are excavation and curatorial traditions, which have further biased the record. This is exemplified by historical artefact collections from sites investigated in the nineteenth and early twentieth century, when the normal archaeological practice was to remove huge quantities of deposit at speed; sieving was rarely employed and consequently, the majority of artefacts and smaller faunal remains were missed entirely during excavation. Today, the emphasis is on recovering and studying every small piece of bone, but this was not the case in earlier excavations when bones which were not regarded as taxonomically identifiable were discarded during fieldwork [94]. This bias is further compounded by curatorial decisions that resulted in the discarding of ‘duplicates’ and bone fragments that were otherwise considered completely unimportant (see Currant, for an Upper Palaeolithic example [95]). These practices have resulted in the inadvertent loss and selective removal of the bulk of unmodified bone tools and retouchers from many archaeological assemblages, which presents a challenge to interpreting and making comparisons of historic and modern collections [94,96,97].

It is important to note that until recently, faunal analysts did not systematically examine all long-bone splinters for taphonomic alterations [98]. As a result, many bone fragments that could have potentially been retouchers were never recorded. Even with systematic collection and examination of every bone fragment, it is still possible for bone retouchers to go unnoted or be misidentified as the products of natural processes [67].

Bacho Kiro Cave in Bulgaria stands out as an early exception to the usual practice of disregarding bone fragments at the excavation stage [99]. Garrod's excavation in 1938 included a detailed examination of undiagnostic bone material, which recognized ‘fragments, chiefly of diaphysis, broken by man, and the majority of these show signs of utilization. (Pl. XXVIII)’ [99, p. 73], including several bone-knapping tools from Layers E and K. Although Garrod acknowledged that smaller, unidentifiable bone fragments were likely discarded, her descriptions and illustrations of the compressors helped alert later excavators to this category of artefact. More extensive excavations in the 1970s by Ginter, Kozłowski and colleagues [100] recovered further examples of retouchers from both Middle and Upper Palaeolithic levels. Ongoing excavations have added to the sample of unshaped bone tools [101]. This work identified retouchers as the only bone tools in Middle Palaeolithic contexts, and a further 44 examples were recognized in the Initial Upper Palaeolithic layers [102].

An additional problem with assessing the record is that minimally used tools can be difficult to distinguish from bones that were chewed by carnivores or bones broken by humans to extract marrow. Thus, even if bone fragments were collected and are well preserved, it can be difficult to separate low-intensity knapping damage from other causes of anthropic or natural modifications [103]. Moreover, the presence and identification of retouchers in archaeological contexts may have been complicated by various post-depositional factors. For instance, the surfaces of bones could have undergone mechanical or chemical post-depositional alterations, leading to additional fragmentation and degradation of cortical surfaces. As a result, the original knapping marks on these bones may have become obscured or even obliterated, making it more challenging to recognize the presence of retouchers and their associated activities.

## 4. Methods

Archaeologists identify osseous knapping tools from characteristic use-wear patterns consisting of parallel gouges and punctiform pits on the surface of bones, antler and teeth [16,22–24]. These marks are the result of the knapping tool making contact with a stone core or the edge of a stone tool during lithic reduction. The knapping process may involve direct percussion with a ‘hammer’, bipolar hammer-and-anvil knapping, pressure flaking with a pointed bone/antler tine or by striking a punch indirectly. Drawing comparisons with experimental examples and making detailed observations of use-wear patterns can aid in reconstructing the way in which these retouchers were manipulated [91]. Furthermore, differences in use-wear have been identified for various factors, such as the use of pressure-flaking versus percussion [104,105] and different types of raw materials, such as obsidian and flint versus quartzite and coarse-grained volcanic rocks [56]. Although most osseous knapping tools had a limited lifespan, the varying degrees of tool use reveal a distinction between expedient or ad hoc knapping tools with minimal scarring, and curated hammers and retouchers that display significant knapping attrition from the extensive use from working multiple lithic tools.

Features of the damage on osseous knapping tools can also help to identify percussors that were used with powerful blows to remove large flakes or work intractable lithic raw materials. Such examples can be recognized by the substantial flaking that often resulted in the percussor shattering during use [20,50,106,107]. Even if knapping tools are absent from an assemblage, different knapping techniques and percussor types are generally identified by flake patterns with characteristic combinations of attributes [108–111].

Long-bone shaft fragments are the most frequently found type of Palaeolithic osseous knapping tool, categorized as ‘retouchers’ and ‘compressors’ (e.g. [20,26,31,48,58]). Nevertheless, a range of other bone types, including ribs, distal ends of humeri, complete limb bones, teeth, and rare examples of antlers modified to make rod-shaped hammers (billets), have been used as knapping tools [103]. The variation in knapping marks can be attributed to several factors, including the type of stone tool being produced, the raw material being worked, the knapping action (direct percussion, indirect percussion, pressure flaking, anvil techniques), the amount of force used, the duration of use, the condition of the bone (fresh versus weathered), and whether preparatory scraping was undertaken to remove periosteum and scraps of flesh from the striking area.

**Figure 1.**
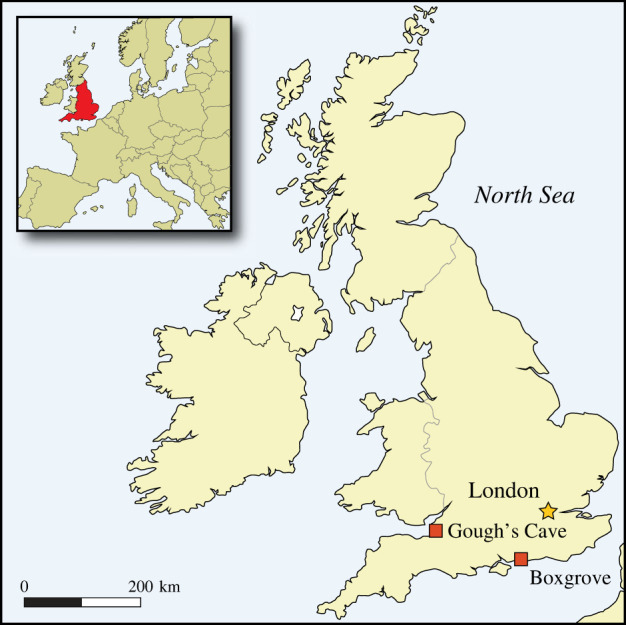
Location of sites.

To strengthen our interpretations of the disputed specimens from Boxgrove and Gough's Cave (figure 1), we employed methods used by Bello and colleagues [58,103,112] for identifying osseous knapping tools. This approach combines the examination of surface features at different scales from the macroscopic (type of osseous element, anatomical location, density of the marks, depth of features and morphological types) to the microscopic level (e.g. microstriations, lithic inclusions).

We employed three-dimensional imaging, computed tomography scanning, scanning electron microscopy (SEM) with elemental surface mapping analysis, and comparisons of archaeological examples with experimental knapping tools used to replicate Palaeolithic stone artefacts and bones modified by known natural taphonomic agencies [113,114]. Initially, the bone surfaces were scanned using a Nikon SMZ-10 binocular microscope up to 40× to locate surface alterations. The topography and microscopic details of the modified surfaces were recorded using a focus variation microscope (FVM)—the Alicona InfiniteFocus G5+ (AIF) optical surface measurement system and a Dino-Lite Edge digital microscope with magnifications up to 140×. For higher resolution images we used a JEOL-IT500 scanning electron microscope, operated in variable pressure mode. The combination of SEM with energy dispersive X-ray spectroscopy (EDX) (Oxford Instrument X-Max 80 Silicon Drift Detector with INCA software) was adopted to obtained elemental analyses of surface features which may contain exogenous elements embedded within the modifications (e.g. microchips within knapping marks).

The effectiveness of this approach lies in its ability to distinguish the often subtle differences between naturally modified bones and implements used as knapping tools, offering a reliable framework for our analysis of the Boxgrove and Gough's Cave bone tool assemblages.

## 5. Results: contentious interpretations of bone tools, carnivore chewing and heavy percussion

This section describes bones from two Pleistocene archaeological sites where the presence of osseous knapping tools is contentious. These examples come from the Lower Palaeolithic (Acheulean) and the latest stage of the Upper Palaeolithic (Magdalenian) and involve conflicting interpretations of the same specimens. Some researchers have identified the modifications on these specimens as carnivore tooth marks [33,115], whereas others have argued that they are actually traces of flint knapping [38,84].

### 5.1. Acheulean flint-knapping tools and carnivores at the Horse Butchery Site, Boxgrove, West Sussex, UK (approx. 0.5 Myr BP)

The Lower Palaeolithic site at Boxgrove is located 10 km from the English Channel coast, in West Sussex, UK (50°52′19′′ N, 0°41′50′′ W, figure 1). During the early Middle Pleistocene, Boxgrove was situated on a chalk sea-cliff, where a rich supply of good quality flint and a resource-rich landscape provided a focus for early hominin flint-knapping and butchery activities. The area was abundant in resources, with mixed woodland on the hills above the site and grasslands and freshwater ponds that developed on the coastal plain due to a drop in sea level towards the end of the Boxgrove interglacial (*ca* 480 kya). Boxgrove is renowned for its prolific early Middle Pleistocene handaxe industry and evidence of large mammal butchery and bone tool use. The handaxe manufacturing technique includes the first evidence for carefully controlled platform preparation coupled with the use of soft hammers to produce thin and highly symmetrical bifaces incorporating a tranchet cutting-tip [34]. The site has also provided the oldest comprehensive evidence of stone tool production using soft tools made of antler and bone [14,38]. These findings, along with the discovery of hominin remains [116–118], greatly enhance the significance of Boxgrove as a key site for gaining insights into human evolution and behaviour at the limits of the occupied world at this time.

Flint-knapping tools have been found at several locations in the Boxgrove quarries. One of these localities is the Horse Butchery Site (Q2 GTP17) where a short-lived land surface preserves a single brief activity event identified from a continuous layer of refitting flint debitage and intermingled butchered remains of a large female horse. The exceptional preservation of the HBS can be attributed to its swift burial under calcareous intertidal muds. The extensive area that has been investigated at the HBS offers valuable insights into the processes involved in manufacturing handaxes and other cutting tools from flint nodules, as well as the meticulous butchering technique used to extract various resources from the carcass. These resources encompassed not only skin, meat, marrow and bone juice, but also bone fragments repurposed as knapping tools (see [35] for a detailed analysis).

The large mammal remains from the HBS were published by Smith [33,119,120]. Smith concluded that hominins had primary access to the horse carcass, which he suggested was acquired by hunting or dispatching an injured animal with a spear. He confirmed earlier observations [32] that the HBS represents a single episode of butchery, during which hominins removed all usable parts of the horse carcass from the site. Smith further concluded that this intensive utilization of the carcass, which had stripped the bones of meat and marrow, explained the limited impact of scavenging carnivores on the residues left by the hominins.

Central to Smith's interpretation of the HBS is an ilium fragment (NHMUK PV M 103080al, field number Q2 GTP17 F278) deriving from the butchered horse. Smith identified marks on this specimen as ‘hominin cut marks overlain by carnivore modifications’ [33, p. 3763]. However, his annotation of the illustration [33, fig. 9, p. 3763] is confusing as the black arrows indicating ‘hominin cut marks’ point to the same set of diagonal incisions (white arrows) that he identifies as ‘carnivore modifications'. Disregarding this confusion, Smith identified the deeper scores that cut across the incisions as carnivore modifications.

**Figure 2.**
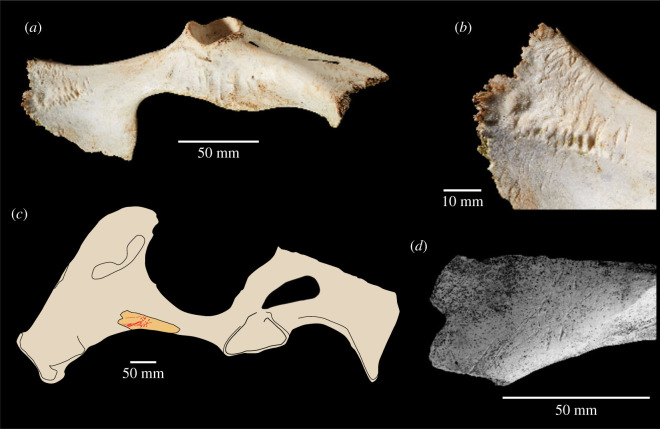
Macroscopic similarity of carnivore chewing marks and knapping marks illustrated by a modern fallow deer innominate chewed by a small carnivore (*a*,*b*) and knapping and scrape marks on a horse innominate fragment (*c*,*d*) from the Boxgrove Horse Butchery Site (NHMUK PV M 103080al, field number Q2 GTP17 F278). The marks on the latter specimens were identified by Smith [33, p. 3763] as ‘hominin cut marks overlain by carnivore modifications'.

The identification of the deeper scores as carnivore tooth marks was based on a macroscopic examination. We note the macroscopic similarity of these marks to illustrations of wolf-chewed caribou ilia illustrated by Binford [8, figs. 3.38, 3.39]. Our comparisons also include a modern innominate of a fallow deer (figure 2) that has been chewed by a smaller canid (probably fox). In these examples, the chewing marks are in the same location as the deeper gouges on the HBS specimen, to which they bear a similarity in their macroscopic features. However, an important morphological difference that rules out carnivore chewing is that the marks on the HBS specimen occur only on one face of the ilium, whereas carnivore chewing leaves tooth marks on both sides of the bone.

**Figure 3.**
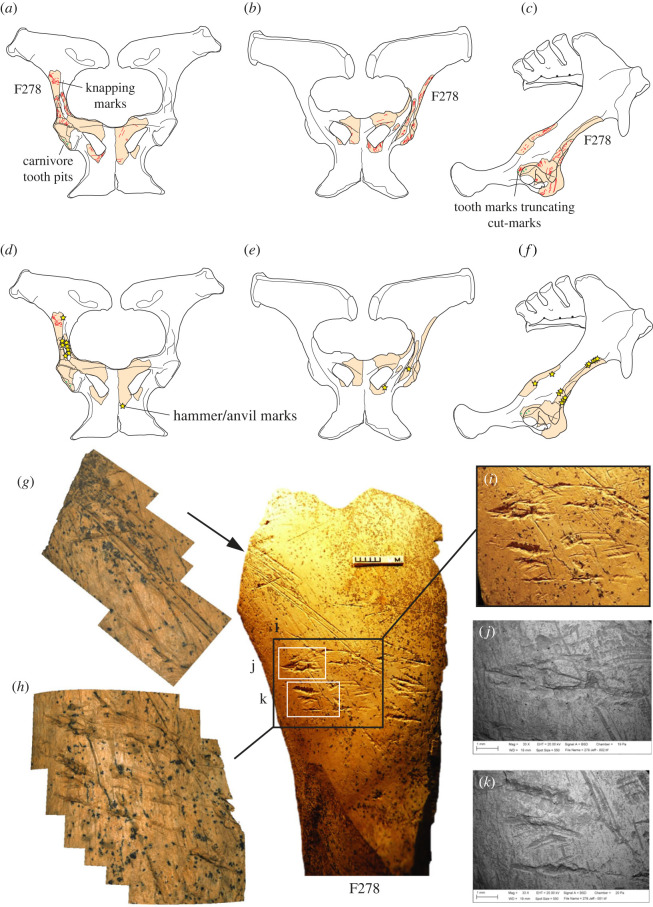
Outline of horse pelvis (*a–f*) from the Horse Butchery Site, showing locations of fragments, cut marks (red lines), hammer/anvil percussion features and carnivore tooth marks (overlying cut marks) on the acetabular rim. (*g–k*) Ilium fragment (NHMUK PV M 103080al, field number Q2 GTP17 F278) used as a knapping percussor with details of scraping marks (*g*) and knapping marks (*i–k*) cutting across the scrape marks (*h*). (*g*,*h*) Three-dimensional Alicona images and (*j*,*k*) SEM images. See figure 4 for comparison of microscopic features of knapping marks on the ilium fragment and tooth marks on the acetabular ‘rim’.

Our interpretation of the alterations is further supported by a microscopic examination of the HBS ilium fragment, which identifies both sets of features as deriving from contact with a stone tool edge. The first set of incisions are broad areas of parallel and sub-parallel marks from scraping with a flint tool with an irregular (e.g. retouched or bifacial) edge (figure 3). We interpret these as focused scraping actions to clean the surface of soft tissue prior to its use as a knapping tool. This probably involved no more than a few scraping actions during which the most prominent asperities along the irregular edge of the stone tool left traces from contact with the bone. Another feature suggestive of scraping are the prominent ‘chattermarks’ that are present within some of the deeper incisions (figure 3). Similar preparatory scraping is observed on several other osseous knapping tools from Boxgrove [34].

**Figure 4.**
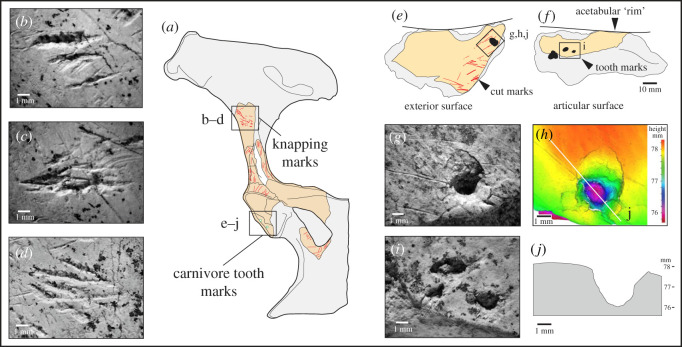
Microscopic differences between knapping marks (*b–d*) and carnivore tooth marks (*g–j*) on fragments of a horse pelvis (*a*) from the Horse Butchery Site. (*b–d*) Overlapping knapping scores in close-up, showing characteristic chipping, angular pits and scores and oblique and transverse microstriations. Knapping marks overlie filleting cut marks. The piece used as a knapping tool is a fragment of the ilium, which was broken intentionally with a hammerstone. (*e–j*) Carnivore tooth marks occur on both sides of the ‘rim’ of the acetabulum (*e–f*). A tooth mark puncturing cut marks (*g*) is illustrated as a topographic model (*h*) and in a cross-section (*j*) generated by the Alicona imaging system.

The marks identified by Smith [33] as carnivore modifications are shorter, deeper scores that cut through the earlier set of scraping marks, at acute angles between 10° and 30°. Observed under low-power magnification, these scores exhibit internal transverse microstriations and chipping of the cortical bone, which is more pronounced on one side of the groove than the other. The scores include smaller oval pits and elongated grooves, but in all cases, they exhibit a consistent set of microscopic features with internal perpendicular microstriations and similar asymmetric V-shaped cross sections (figures 3*h–k* and 4). Although no flint microchips were observed embedded in the impact features, the overall characteristics of the grooves are entirely consistent with the bone fragment having been used to sharpen or rejuvenate the edge of a stone tool edge with as few as 20 light blows.

The ilium fragment also exhibits pre-existing features including filleting cut marks and hammerstone percussion marks. The distribution of hammerstone blows, as shown in figure 3*d–f*, indicates that the breakage of the pelvis was aimed at accessing the spongy bone to extract bone juices, whereas the intensity of hammerstone marks along the edge of NHMUK PV M 103080al (field number Q2 GTP17 F278) suggests that further chipping was undertaken on this piece to shape this part of the ilium in order to produce a tool with thick cortical bone that could be held and manipulated easily [38]. The fact that the bone fragment was selected from among pieces of the shattered pelvis suggests that it was modified and used as a knapping tool at a relatively late stage in the butchery sequence. This supports the interpretation that this ad hoc knapping tool was used to resharpen a lithic edge that was becoming blunt during the final stages of the butchery of the horse.

**Figure 5.**
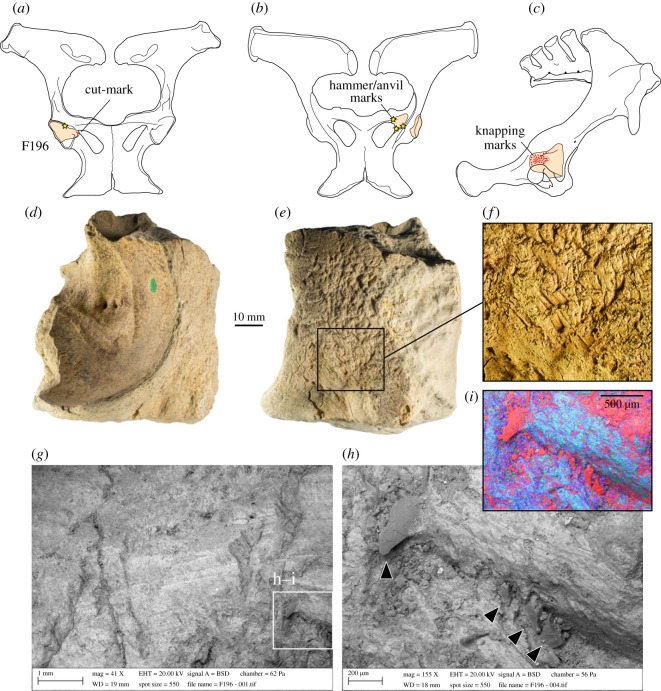
An acetabulum fragment (NHMUK PV M 103079) from a second horse individual used as a knapping percussor at the Horse Butchery Site: (*a–c*) outline drawings showing the location of piece, hammer/anvil marks from shaping the hammer and knapping marks; (*f*) close-up of knapping marks; (*g–h*) SEM images illustrating gouges, microstriations and embedded flint chips (black arrows); (*i*) EDX image mapping the elemental composition of the bone surface (blue = calcium) and highlighting the embedded flints (red = silicon).

Our analysis of the HBS assemblage identified another complete knapping percussor NHMUK PV M 103079 (field number Q2 GTP17 F196; figure 5) and four articular and six cortical bone fragments from shattered knapping tools (table 1). Eight of these specimens are described in Parfitt & Bello [38], and four additional pieces were identified subsequently. Although Smith identified bones used as ‘lithic retouchers’ from different localities at Boxgrove [33, fig. 12, p. 3764], it is unclear whether he identified the modifications of the additional HBS examples as knapping marks, carnivore chewing or butchery marks.

**Table 1.** Revised list and features of organic knapping tools from the Horse Butchery Site, Boxgrove. ✓, modification present; cut, butchery cut mark; Sh, shaping; Sc, scraping; K, knapping damage; Br, breakage during use; Mf, breakage during marrow processing.

NHM accession number (NHMUK PV M)	site find no. (Q2 GTP 17)	taxon	anatomical part	impact damage from ‘shaping’	cut marks	scrape marks	knapping marks	probable sequence of alterations
103080al	F278	*Equus ferus*	innominate (ilium shaft)^a^	✓	✓	✓	✓	Cut → Sh → Sc → K
103079	F196	*Equus ferus*	innominate (acetabulum)^b^	✓	✓		✓	Cut → Sh → K
103076	F457	large mammal	compact bone				✓	K → Br
103075	F476	large mammal	compact bone				✓	K → Br
103080t	F738	large mammal	compact bone				✓	K → Br
103074	F467	large mammal	Compact bone				✓	K → Br
103077	F363	large mammal	articular bone				✓	K → Br
103078	F869	large mammal	articular bone				✓	K → Br
103080bf	F436	*Equus ferus*	radius (shaft frag.)		✓	✓	✓	Cut → Sh → Sc → K → Mf
106362	F569	*Equus ferus*	humerus (distal epiphysis frag.)				✓	K → Br
103080cf	F571	*Equus ferus*	humerus (distal epiphysis frag.)				✓	K → Br
106363	F760	large mammal	compact bone				✓	K → Br

^a^Horse individual 1 (butchered on site).

^b^Horse individual 2 (transported knapping tool).

A second complete osseous knapping tool identified at the HBS is a horse acetabulum percussor NHMUK PV M 103079 (figures 4 and 5) that exhibits prominent and easily recognizable knapping marks. The specimen comprises the majority of the right acetabulum, which notably belongs to a different horse individual whose remains were not otherwise present at the site. Hammerstone percussion marks, similar to those observed on the innominate fragment (NHMUK PV M 103080al), have been identified on this specimen as well, indicating how it was reduced to produce a hammer that could be conveniently held by the knapper. This artefact is interpreted as a portable knapping tool that was brought to the site along with flint nodules and used during the initial stages of handaxe production to shape and thin the stone tools [38].

The pieces interpreted as fragments of broken knapping tools are small bone fragments (18–68 mm), but all bear the distinctive combination of overlapping pits and scores with microstriations, some of which contain embedded flint chips (table 2). Marks on some of these specimens were only visible under the binocular microscope when the specimen was illuminated at a low angle, with the lighting source positioned precisely in the right direction. We interpret these as fragments from knapping tools that broke during use, or they are from bones that were smashed to extract marrow or bone juice after they had served their function as knapping tools. One of the pieces comes from the shaft of a horse radius (NHMUK PV M 103080bf, field number Q2 GTP17 F436), which forms a refitting set of eight other pieces from the distal half of the diaphysis. The fragment and adjacent conjoining pieces have preparatory scraping marks that are overlain by knapping marks concentrated along the posterior–lateral angle of the shaft. The radius was used as a knapping hammer when it was complete, and the breaks were the result of subsequent percussion aimed at extracting the marrow. This impact point is located higher up on the shaft; this blow initiated spiral fractures which truncate the knapping and scraping marks and opened the marrow cavity. This is an example of a long bone from the horse that was used as a knapping tool to rejuvenate a flint-cutting tool as the horse was being disarticulated and filleted.

**Table 2.** Summary of knapping features on bones used as percussors at the Horse Butchery Site, Boxgrove. ✓, feature present; —, not possible to score due to poor surface preservation, small fragment size or features obscured by sediment. Use intensity: C and S, concentrated and superposed; C, concentrated; D, dispersed (see van Kolfschoten *et al*. [19] for details).

NHM accession number (NHMUK PV M)	site find no.	taxon	anatomical part	number of knapping areas	preparatory scrape marks	embedded flint	pit (P), score (S), gouge (G)	internal striations	tool-edge scratches	use intensity
103080al	F278	*Equus ferus*	innominate (ilium shaft)	1 of 2	✓		S	✓	✓	C
103080al	F278	*Equus ferus*	innominate (ilium shaft)	2 of 2	✓		P > S	✓	✓	D
103079	F196	*Equus ferus*	innominate (acetabulum)	1		✓	S, G > P	✓		C and S
103076	F457	large mammal	compact bone	broken		✓	S, P	✓		C and S
103075	F476	large mammal	compact bone	broken	—		S, G	—	—	D
103080t	F738	large mammal	compact bone	broken		✓	S, G	✓		C
103074	F467	large mammal	compact bone	broken	—	✓	S	—	—	
103077	F363	large mammal	articular bone	broken	—	✓	S	—	—	C
103078	F869	large mammal	articular bone	broken	—	✓	P	—	—	
103080bf	F436	*Equus ferus*	radius (shaft frag.)	1	✓	✓	P, S	✓	✓	D
106362	F569	*Equus ferus*	humerus (distal epiphysis frag.)	broken			P	✓		C
103080cf	F571	*Equus ferus*	humerus (distal epiphysis frag.)	broken			P, S	✓		C
106363	F760	large mammal	compact bone	broken	—		S	—		C and S

**Figure 6.**
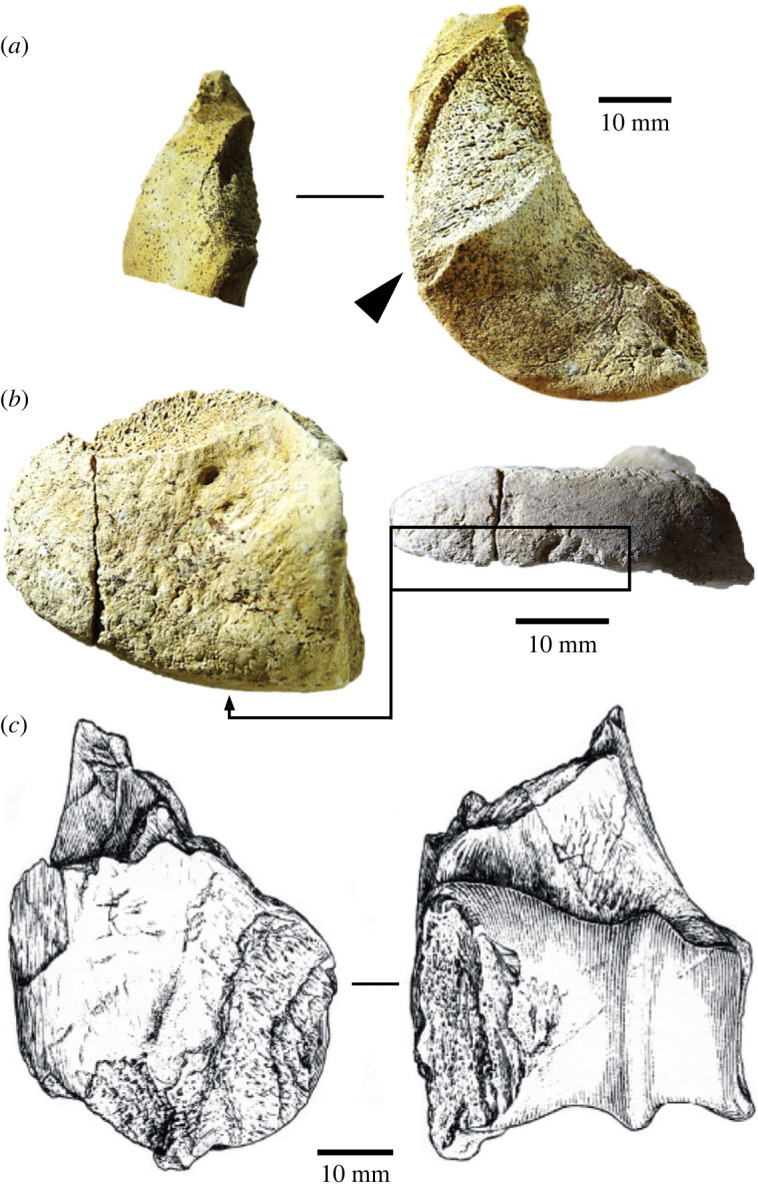
Long-bone epiphysis fragments showing damage characteristic of flint-knapping tools: (*a*,*b*) flaked distal articular ends of humeri from the Horse Butchery Site ((*a*) NHMUK PV M 103080cf, field number Q2 GTP17 F571; (*b*) NHMUK PV M 106362, field number Q2 GTP17 F569), showing knapping damage (in (*a*), arrow indicates the direction of the impact that detached a flake of bone from the side of the condyle), and (*b*) parallel gouges from contact with a stone tool edge during knapping (area bounded by the black box); (*c*) heavy impacts during knapping on this medial epicondyle of a cervid distal humerus (NHMUK PV UNREG 4200, field number Q1/B F4983) from the Boxgrove Hominin Site (Q1/B) would have resulted in flakes similar to those from the Horse Butchery Site (drawing by Julian Cross).

**Figure 7.**
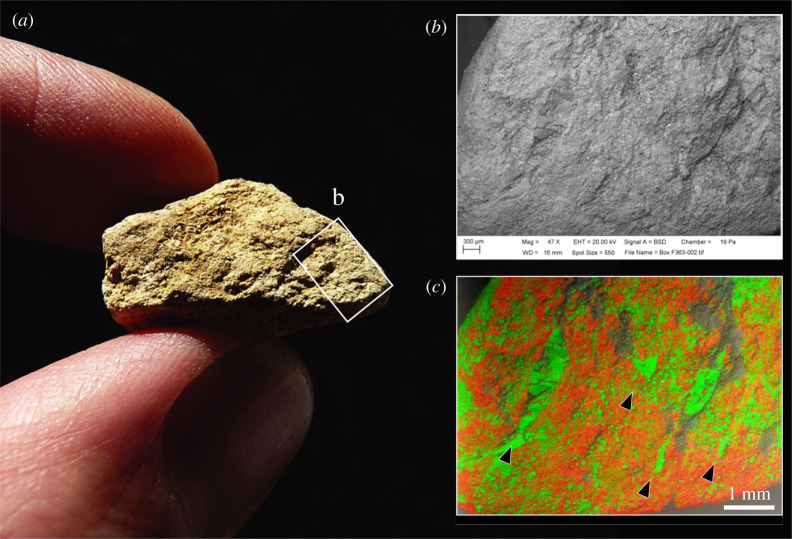
Degraded articular bone fragment (NHMUK PV M 103077, field number Q2 GTP17 F363) from the Horse Butchery Site interpreted as a fragment from a knapping hammer (*a*). A feature of this piece is the parallel zones of embedded fractured flint fragments, which is typical of knapping areas on the articular ends of more complete knapping hammers from Boxgrove (e.g. figure 6*c*). The embedded flint chips are illustrated in SEM (*b*) and SEM–EDX (*c*) images. In the SEM–EDX image, the flint chips are green (with dispersed silica matrix), and the bone (calcium) is red; black arrows mark larger zones of embedded, crushed flint.

The articular fragments (figures 6 and 7) include two particularly informative pieces (NHMUK PV M 106362, field number Q2 GTP17 F569 and NHMUK PV M 103080cf, field number Q2 GTP17 F571). These are ‘flakes’ from the distal condyles of the right and left humeri, which are similar to the flake scars on several of the more complete distal humeri from the Waterhole Site in Quarry 1 (e.g. [14, fig. 45]). The flake removals on the Quarry 1 examples are associated with intense battering on the distal condyles of cervid and bovid humeri, which were used as knapping hammers. The flakes from the HBS also have traces of knapping damage, suggesting that these are fragments that flaked off the margins of the condyles during the knapping process. One of the other articular fragments is too small to identify to bone element. The surface is also degraded, but SEM–EDX shows that there are parallel shattered flint chips embedded in the spongy bone (figure 7), which is a characteristic feature of the knapping zones on the distal humeri from the Waterhole Site (e.g. figure 6*c*).

A second aspect of Smith's interpretation of the HBS that deserves comment relates to his suggestion for a limited impact of carnivore scavenging on the assemblage. Our analysis, in contrast, reveals compelling evidence that carnivore scavenging exerted a significant influence on the composition of the bone assemblage. Despite the low incidence of carnivore tooth marks, found on only 1% of bones from the HBS, the presence of these marks, coupled with the discovery of a hyaena coprolite in the western area of the site, conclusively establishes scavenging carnivore activity after the site was abandoned by hominins. Hyaena scavenging would also explain one of the anomalous features of the assemblage composition, namely the low representation of fragments of spongy bone relative to cortical fragments. This pattern is consistent with experimental butchery sites where marrow-fractured bones have been left overnight in an area visited by spotted hyaenas [121]. In these situations, the hyaenas ate the spongy bone fragments, but the shaft fragments were largely ignored, and none were tooth-marked. Furthermore, experiments with captive hyaenas feeding on marrow-fractured bones have shown that they selectively remove spongy bones either by consumption on site or by carrying them to another feeding location, resulting in a similarly low incidence of carnivore tooth marks on the remaining bone fragments and a depletion of spongy bones relative to cortical fragments [122]. These observations are consistent with key features of the HBS bone assemblage, notably the under-representation of cancellous bone relative to cortical pieces, as well as the low overall incidence of carnivore chewing marks on the shaft fragments.

### 5.2. Horse phalanges with percussion features and carnivore chewing from Gough's Cave, Somerset, UK (Magdalenian, approx. 15 000 years BP)

Gough's Cave (51°16′54′′ N, 2°45′55′′ W, figure 1) is situated in southwest England, specifically at the entrance of Cheddar Gorge at the foot of the Mendip Hills. In the late nineteenth century, a significant amount of the cave's infill was removed to improve tourist access, and from 1927, archaeologists conducted intermittent excavations in the cave. The most extensive excavations occurred during the periods 1927–1934 and 1949–1951, and in 1986–1982 when a small remnant of Lateglacial sediment was excavated by a team from the Natural History Museum (London) and Nottingham University. These excavations recovered a rich Late Upper Palaeolithic (Magdalenian) flint industry along with butchered human and other mammal remains, as reported in studies by Currant *et al.* [123], Jacobi [124] and Donovan [125]. Ultra-high-resolution radiocarbon dating of butchered bones and human remains suggests that the Magdalenian occupation of Gough's Cave occurred during a brief period of arctic conditions at the end of the Late Pleniglacial, specifically Greenland Stadial 2.1a, just before the onset of the Late Glacial warming phase, Greenland Interstadial 1e [126].

The Lateglacial faunal assemblage is dominated by butchered horse bones [95,127] and an extensive assemblage of human remains resulting from cannibalistic rituals involving the production of ‘skull-cups’ [115,128–131]. Additionally, the site contains an array of interesting artefacts, including perforated reindeer antlers, a mammoth ivory javelin head, awls, needles and needle blanks, fox tooth pendants, seashells, incised ivory, amber and engraved pebbles [103,132,133]. The available evidence suggests that Magdalenian family groups used the cave as a shelter during short-lived seasonal visits and were likely involved in hunting migrating herds of horses and red deer [124].

The Gough's Cave bone assemblage has been the subject of several taphonomic analyses, each providing valuable insight into the site's occupation history. The first detailed study was conducted by Parkin *et al*. [127], who analysed butchery marks on the bones and identified specialized processing of horse limbs to extract sinews and tendons. They also examined the spatial distribution of the bones and discussed patterns of carnivore scavenging. A subsequent work by Andrews & Fernández-Jalvo [115] compared modifications in the human and non-human bone assemblages, including material excavated between 1989 and 1992. Recently, a paper by Bello *et al*. [84] identified two teeth and seven bones within the same collection with previously unrecognized knapping marks.

These studies highlight discrepancies in the taphonomic interpretation of the Gough's Cave faunal assemblage. To identify possible reasons for such discrepancies, we compare the results of our new analysis of the incidence of cut marks, percussion damage, and carnivore chewing marks on the horse phalanges (tables 3–5) with the interpretations of Parkin *et al*. [127] and Andrews & Fernández-Jalvo [115]. We discuss the discrepancies between our results and those of the earlier studies.

**Table 3.** Counts of Gough's Cave Magdalenian horse phalanges examined in three taphonomic studies. Collections: NHM, Natural History Museum, London; CCM, Cheddar Caves Museum; WM, Wells Museum; TCM, Taunton Castle Museum.

study	phalanx I	phalanx II	phalanx III	total	collection
Parkin *et al*. [127]	19	12	18	49	NHM^a^, CCM, WM, TCM
Andrews & Fernández-Jalvo [115]	20	11	18	49	NHM^a,b^
this study	22	14	20	56	NHM^a,b^

^a^Parry (1929–1932) excavation.

^b^NHM excavation.

**Table 4.** Comparison of taphonomic modifications affecting horse phalanges from Gough's Cave recorded by Parkin *et al*. [127], Andrews & Fernández-Jalvo [115] and this study. Percentages in italics (columns 6 and 7) are plotted in figure 8. NP, counts not published. Collections studied: Parry excavation material in NHM; combined Parry and NHM excavation material—note three specimens in the third phalanges collected by Parry are covered in concreted sediment; these specimens are excluded from the sample (*) and mean values calculated for our study.

study	Parkin *et al*. [127]		Andrews & Férnandez-Jalvo [115]		this study	
sample	Parry excavation	Parry excavation	Parry and NHM excavation	Parry and NHM excavation	Parry^1^ and NHM excavation^2^	Parry^1^ and NHM excavation^2^
anatomical element	phalanx I	phalanx I–III	phalanx I	phalanx I–III	phalanx I	phalanx I–III
number of specimens	19	49	20	49	Parry^1^ = 19 Parry and NHM^1+2^ = 22	Parry^1^ = 50 (47*) Parry and NHM^1+2^ = 56 (53*)
cut-marked	13 (68.4%)	29^a^ (59.2%)	NP	29 (59.1%)	12 + 1? (68.4%^d^; 63.2%^e^)^1^	28 + 4? (68.1%^d^; 59.5%^e^)^1*^
					14 + 1? (68.2%^d^; *63.6*%^e^)^1+2^	33 + 5? (71.6%^d^; *62.3*%^e^)^1+2*^
carnivore chewed	7 (36.8%)	9 (18.4%)	2^b^ (10%)	2 (4.1%)	8 (42.1%)^1^	9 + 2? (23.4%^d^; 19.1%^e^)^1*^
					8 (*36.4*%)^1+2^	9 + 2? (20.7%^d^; *17.0*%^e^)^1+2*^
cut-marked and carnivore chewed	5 (26.3%)	5 (10.2%)	NP	NP	4 + 1? (26.3%^d^; 21.0%^e^)^1^	4 + 2? (12.7%^d^; 8.5%^e^)^1*^
					4 + 1? (22.7%^d^; *18.2*%^e^)^1+2^	4 + 2? (11.3%^d^; *7.5*%^e^)^1+2*^
percussion features	0	0	14^c^ (70%)	27 (55.1%)	1 + 2? (13.6%^d^; *4.5*%^e^)^1+2^	1 + 2? (5.6%^d^; *1.9*%^e^)^1+2*^

^a^In Parkin *et al*. [127], there is a discrepancy between the number of cut-marked first phalanges recorded in the appendix (*n* = 29) and the 30 cut-marked specimens shown in figure 8.

^b^Counts of carnivore-chewed specimens derived from text in Andrews & Férnandez-Jalvo [115].

^c^Number of percussion-marked first phalanges calculated by subtracting the number of percussion-marked phalanges II–III recorded by Andrews & Férnandez-Jalvo [115] from the total number of phalanges examined in their study.

^d^Percentage including specimens where the identification of features is uncertain.

^e^Percentage excluding specimens where the identification of features is uncertain.

**Table 5.** Butchery marks and carnivore chewing on horse metapodials and phalanges from Gough's Cave.

modification	metapodial	phalanx		
	(*n* = 14)	phalanx I (*n* = 22)	phalanx II (*n* = 14)	phalanx III (*n* = 20)
cut only	5	9	9	8
(?) cut only			1	2
retoucher only	1			
cut and retoucher	2	1		
cut and chewed	3	4		
cut and (?) chewed				2
(?) cut and chewed		1		
chewed only	1	3		1
neither cut nor chewed	2	3	3	4
embedded in matrix, weathered or surface otherwise degraded				3

To enable a meaningful comparison of the three datasets, however, it is important to note that they differ slightly in terms of the specimens examined (table 3). Andrews & Fernández-Jalvo [115] combined specimens from both the 1927–1931 excavations housed in the NHM and the more recent excavations conducted between 1989 and 1992. By contrast, Parkin *et al*. [127] only analysed the 1927–1931 collection at the NHM along with some additional specimens in other museums that were not seen by Andrews and Fernández-Jalvo.

**Figure 8.**
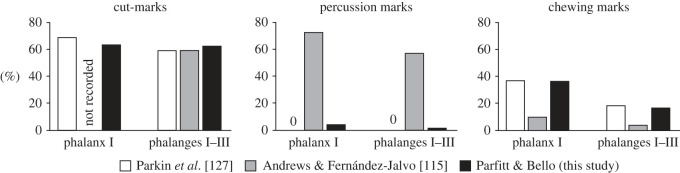
Comparison of cut mark, percussion damage and carnivore chewing prevalence on Gough's Cave horse phalanges, as recorded by Parkin *et al*. [127], Fernández-Jalvo & Andrews [115] and this study (Parfitt and Bello).

To ensure comparability with our own results, we have provided two sets of counts: the first for the 1927–1931 sample at the NHM, which can be directly compared with the data presented by Parkin *et al*. [127]. The second tabulation combines this sample with the material from the 1989–1992 investigations, making the counts comparable with those of Andrews & Fernández-Jalvo [115]. To allow a more detailed analysis, we have also tabulated the results separately for the first phalanx and the full set of phalanges (I–III). By doing so, we can identify any patterns or discrepancies in the distribution of cut marks, percussion damage and carnivore chewing marks on the horse phalanges across the different datasets (table 4 and figure 8).

**Figure 9.**
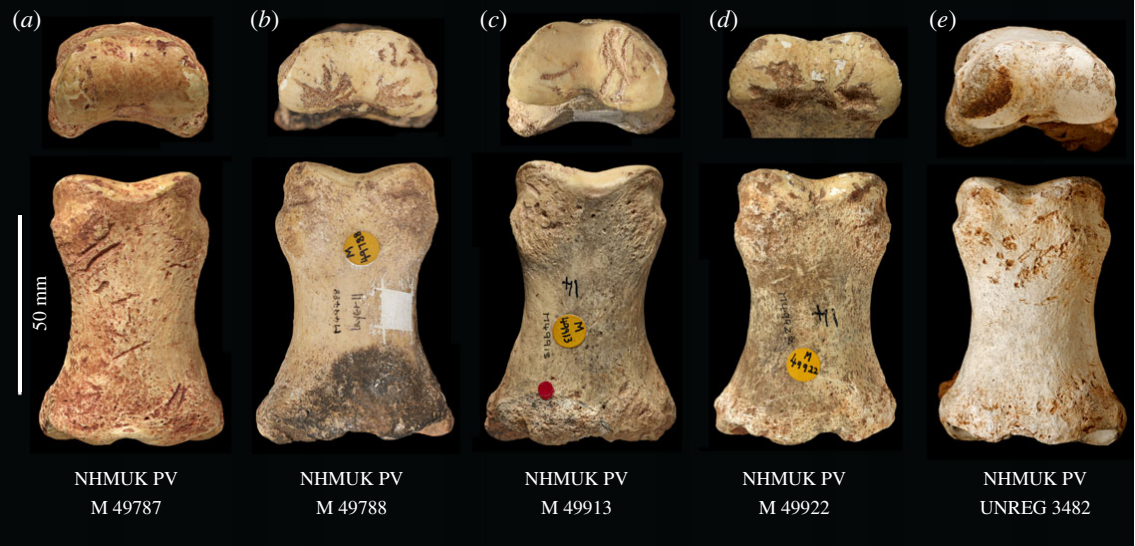
Carnivore chewing marks (*a–d*) and knapping marks (*e*) on horse first phalanges from Gough's Cave (dorsal and distal views). In an earlier study, the marks on the phalanges were identified as heavy percussion damage [88]. See figure 10 for details of the chewing marks (figure 10*a*–*e*) and knapping marks (figure 10*f*–*j*).

In the first study, Parkin *et al*. [127] identified cut marks on 59% of the phalanges and a relatively high incidence of carnivore gnawing (up to 37% of the first phalanges); no examples of phalanges marked by percussion were noted in their publication. The later study by Andrews & Fernández-Jalvo [115] recorded a similar frequency of cut-marked bones, but an opposing pattern with a higher incidence of percussion damage (up to 70%) and a considerably lower prevalence of carnivore chewing amounting to 10% of the first phalanges, which falls to 4.1% when the other phalanges are included in the count (table 4 and figure 8). Despite the high incidence of heavy percussion damage recorded by Andrews & Fernández-Jalvo [115], it is notable that nearly all the phalanges are complete (figure 9).

**Figure 10.**
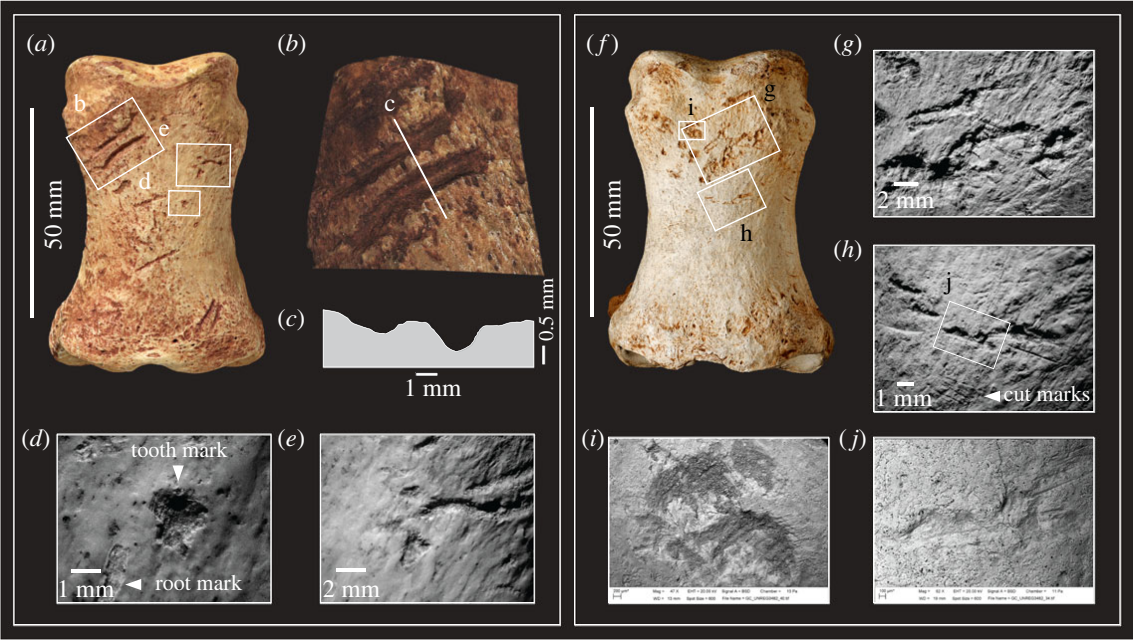
Microscopic differences between carnivore tooth marks (*a–e*) and knapping marks (*f–j*) on horse phalanges from Gough's Cave. Carnivore tooth marks on horse phalanx NHMUK PV M 49787 include scores (*b*–*c*,*e*) and pits (*d*,*e*). The smooth-bottom scores with U-shaped cross sections are shown in a surface model (*b*) and a section generated by the Alicona imaging system. (*f–h*) Overlapping knapping marks exhibit angular chipping and oblique and transverse microstriations on horse phalanx NHMUK PV UNREG 3482. Similar microabrasions associated with pits and scores are visible in SEM micrographs (*i*,*j*) and are interpreted as tool-edge scratches (*sensu* [19]).

Our results are more closely aligned with the results of the Parkin *et al*. study [127], notably the equivalent levels of carnivore gnawing (36%) and the cut mark (63%) prevalence (figure 8 and table 4). Our results diverge, however, in our recognition of percussion damage on the phalanx NHMUK PV UNREG 3482 (the knapping percussor), and possible percussion breakage of two additional phalanges (NHMUK PV M 49945 and NHMUK PV UNREG 3522). The first of these is arguably the best example of a retoucher from Gough's Cave, with comparable examples known from several sites on the European mainland [84,106]. The knapping (percussion) damage is concentrated on the dorsal surface towards the distal end and is consistent with the phalanx having been used in direct percussion to knap flint tools (figure 10*f–j*). NHMUK PV UNREG 3482 was not recognized as a knapping tool prior to the study by Bello *et al*. [84].

The breakage of the other two phalanges was described in detail by Andrews & Fernández-Jalvo [115], who observed that NHMUK PV M 49945 ‘has the proximal end removed by heavy percussion impacts. A flake has been removed on the medial edge, and the shaft is cracked both longitudinally and transversely with percussion marks on the broken edge.’ In our view, the cause of the breakage of this specimen is difficult to determine. Although the bone is marked by carnivore tooth pits and scores (one of which is associated with a fracture surface) it seems unlikely that fractures could have been inflicted by a relatively small dog, which is the most abundant carnivore in the Gough's Cave assemblage, although it may have been within the capabilities of a large wolf that is a rare component of the Gough's Cave faunal assemblage (e.g. Longleat House, ex. Gough's Cave Museum—maxilla 1. 2/17, mandible 1. 2/15). Significantly, we cannot discern any impact features on this phalanx, and it is possible that the bone was crushed in a rock fall or by sediment pressure when the phalanx was buried but still in a relatively fresh condition. The other fractured first phalanx (NHMUK PV UNREG 3522) is missing part of the proximal end, but again it is difficult to discern the cause of breakage.

Andrews & Fernández-Jalvo [115] reported a high percentage of percussion-damaged phalanges without providing an explanation for these features. Marrow processing can be excluded as all but one of the 54 phalanges are complete. The complete horse phalanges from Gough's Cave can be contrasted with other Magdalenian examples that have been fractured to extract marrow and grease, which have breaks that split the phalanges and expose the internal cavity and spongy bone, as seen in examples from Gönnersdorf (Germany) described by Street & Turner [134]. Similarly, damage from disarticulation and processing of the feet for sinews can be excluded, as this activity at Gough's Cave was undertaken with precise cutting of joint capsules and carefully placed cuts at attachment points of the ligaments and tendons, as noted by Parkin *et al*. [127].

An alternative interpretation for the high percentage of phalanges with percussion damage identified by Andrews & Fernández-Jalvo [115] is that they included carnivore tooth marks in their percussion category (figures 9 and 10). This interpretation is supported by their descriptions and illustrations of two phalanges (NHMUK PV M 49787 and NHMUK PV M 49958) [115] that they identify as having carnivore tooth marks. The marks on these specimens are similar to the scores and pits on phalanges that they attribute in other specimens to damage from ‘very heavy percussion’ [115, fig. 7b, p. 68]. Additional comparisons of marks on phalanges interpreted as heavy percussion damage by Andrews & Fernández-Jalvo [115] are provided in fig. 91 and by Bello & Parfitt [103], who illustrate further examples of carnivore-chewed bones and knapping percussors from Gough's Cave.

## 6. Discussion

### 6.1. Acheulean flint-knapping tools and carnivores at the Horse Butchery Site, Boxgrove: implications

The significance of the Boxgrove handaxes lies in the exceptional abundance of well-preserved specimens found at the site, combined with the burial conditions that have preserved a succession of archaeological horizons deposited within relatively short time frames. These assemblages encompass various contexts, ranging from the rapid burial of single butchery sites, like the HBS, incorporated in the intertidal muds to the development of landsurfaces and waterholes over a span of possibly no more than a few hundred years [135]. The Boxgrove handaxes are now being used as a ‘standard’ for comparisons and discussions on the meaning of morphological variation and standardization in Acheulean handaxes. Factors under investigation include the influence of knapping skill, raw material, reduction method and intensity, and handaxe function. To tackle these challenges, researchers have been employing advanced statistical analyses, as demonstrated in papers by García-Medrano *et al*. [136,137]. These new analytical approaches are providing a basis for comprehensive discussions regarding the importance of mental templates, levels of cognition, the extent of planning capabilities, and the social environments that facilitated learning in Middle Pleistocene hominins [34,138–147].

A recent study by García-Medrano *et al*. [148] has identified ‘idiosyncratic’ features (e.g. ‘tranchet’ finishing of the tip) in the manufacture of the Boxgrove handaxes within one regional handaxe technological group in western Europe. Moreover, the study identified regional innovations in handaxe technology through time that are linked with local traditions in their manufacture. The study suggests that these regional trajectories of technological evolution imply that cultural transmission took place over extended periods of time, perhaps lasting for tens to hundreds of thousands of years (cf. [149]). Several studies (reviewed by Liu *et al*. [147]) have highlighted the particularly high level of knapping skill involved in the production of the Boxgrove handaxes. The level of difficulty involved in thinning the handaxes is compounded by the preference for broad, generally highly symmetrical ovate-style handaxes. Manufacturing such handaxes required the knapper to undertake carefully controlled platform preparation combined with the striking of longer flakes with great precision to achieve the desired thinning without breaking the tool.

How do the knapping tools factor in this discussion? Firstly, it is significant that the knapping Boxgrove toolkit included a range of percussor types, including hard hammers of cortical flint nodules and beach pebbles, as well as different types of osseous percussors [35]. The latter include bone-knapping tools (shaft splinters) used and quickly discarded on the spot, as well as transported osseous hammers (often complete limb bones or epiphyses), some purposefully shaped by percussion and scraping and then curated for later use. Included in this nascent bone technology are the earliest antler knapping hammers of a type that until recently were known only from Upper Palaeolithic contexts in western Europe.

Secondly, lithic refitting studies show that the switch from hard hammers to soft hammers during the production of the Boxgrove handaxes occurred early in the knapping sequence after the outer cortical ‘rind’ had been removed by a hard hammer [109]. The use of a variety of percussors during the shaping and thinning stages allowed for greater control in flake removals, which is one of the key factors in the high degree of shaping intensity and control that resulted in the finely crafted Boxgrove handaxes. This shift in hammer types was critical to the production of the handaxes and demonstrates the ability of the Boxgrove flint-knappers to select, modify and manipulate osseous knapping tools in specific ways to suit various knapping tasks. Turning to the specific example of the HBS knapping tools, this assemblage is important because it includes remnants of osseous tools (i.e. shattered fragments of knapping tools) that are rarely recovered or recognized in Lower Palaeolithic contexts.

Regarding bone tool production methods, the knapping hammers include at least one example (the acetabulum NHMUK PV M 103079) that was transported to the site after having been deliberately shaped to produce a percussor with a specific form and function in mind. The acetabulum exhibits extremely heavy utilization, reflecting intensive and possibly recurrent use that implies its curation over time. This tool is particularly informative as it embodies long-term planning and careful manufacture to impose shape on an unwieldy pelvis, with the aim of improving ergonomic properties and transportability.

A second type of bone percussor is exemplified by a bone splinter (the ilium fragment NHMUK PV M 103080al, field number Q2 GTP17 F278) selected from broken and butchered bones at the HBS. This was chosen because its shape and size made it suitable for use as a knapping tool with only minimal modification (scraping and chipping). This tool was used during a later stage in the processing of the carcass, probably as a re-sharpening tool that was discarded after a short period of use. The relatively small size of the knapping marks suggests the damage was the result of light taps or pressure against a lithic tool edge.

**Figure 11.**
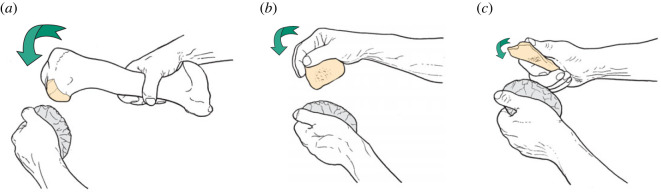
Techniques of shaping and resharpening handaxes with bone hammers. Reconstructions illustrating how soft hammers at the Horse Butchery Site were used with different amounts of force (indicated by arrow size) to shape and/or resharpen handaxes: (*a*) humerus used as a knapping hammer (identified from flaked fragment, NHMUK PV M 103080cf, field number Q2 GTP17 F571); (*b*) acetabulum percussor (NHMUK PV M 103079, field number Q2 GTP17 F196); and (*c*) ilium percussor (NHMUK PV M 103080al, field number Q2 GTP17 F278).

The third type of percussor can be identified from the articular pieces, at least two of which come from the distal ends of humeri (NHMUK PV M 106362, field number Q2 GTP17 F569 and NHMUK PV M 103080cf, field number Q2 GTP17 F571). These were used with much greater force that resulted in the percussors chipping during use. Figure 11 reconstructs the way in which these bones were used as knapping percussors.

The final group includes long-bone shaft fragments. Of these, the most informative is a radius fragment (NHMUK PV M 103080bf, field number Q2 GTP17 F436), which forms part of a refitting set with eight other fragments. The area with the knapping marks has been scraped, and the piece was used with relatively gentle taps rather than forceful blows. Use as a knapping percussor was evidently undertaken before the radius was cracked open to extract marrow (the scraping marks are truncated by a spiral fracture emanating from an impact notch). It is possible that other osseous knapping tools, such as antler hammers, were also used at the site, but it is likely that they were taken away with the handaxes and meat, and therefore not found in the assemblage.

**Figure 12.**
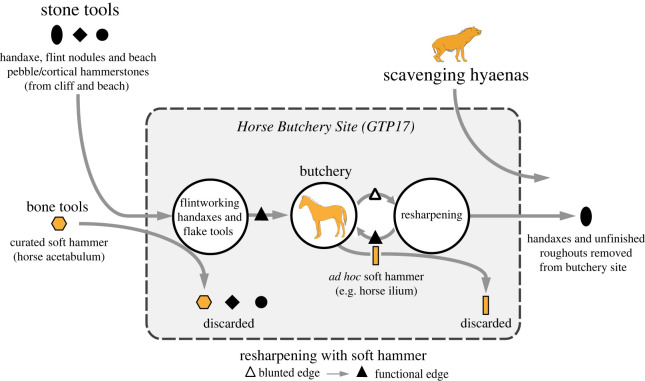
Model integrating the lithic chaîne opératoire with knapping tools and activities undertaken at the Horse Butchery Site.

Although interpreting how the bone-knapping tools were modified and used can be gleaned from examining individual specimens, a more complete picture emerges with the inclusion of the distribution patterns of lithic debris and refits. This approach forms the foundation for integrating the HBS osseous knapping tools into the broader lithic chaîne opératoire, as depicted in figure 12.

**Figure 13.**
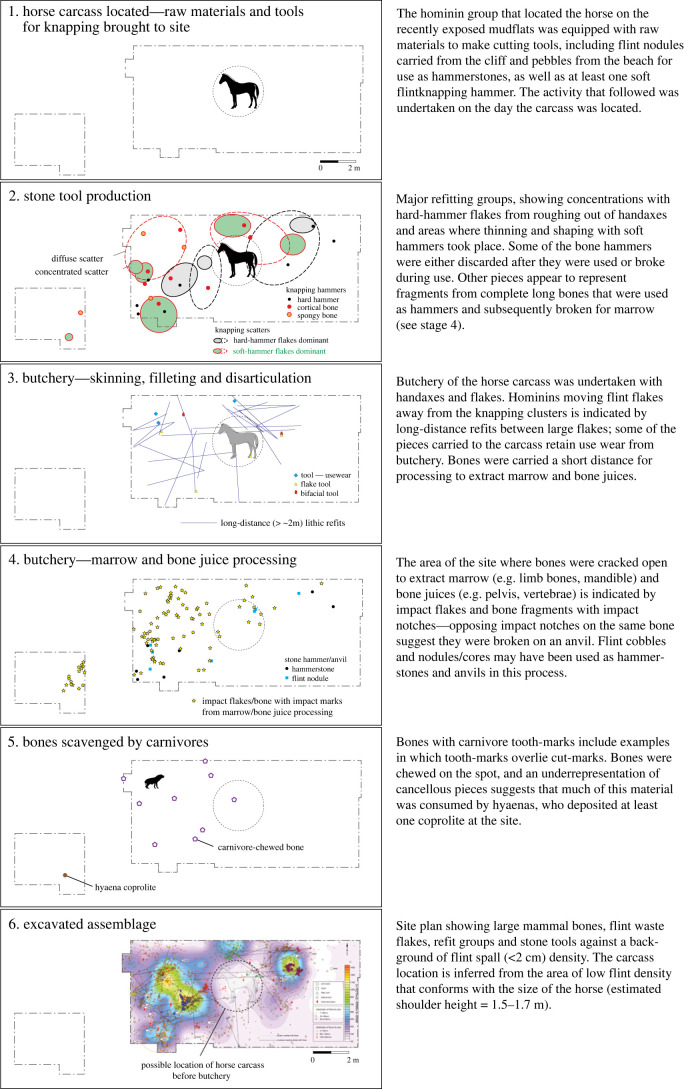
Plans of the Horse Butchery Site illustrating the sequence of events associated with the butchery of the horse carcass.

Taken together, the results from the meticulous excavation of the HBS and analysis of the spatial distribution of artefacts and bones, combined with the detailed taphonomic study of the faunal remains [35] have resulted in an exceptionally comprehensive reconstruction of the chronological sequence of events that likely transpired at the butchery site within a single day. Figure 13 provides a summary of the organization and sequence of activities and behaviour that can be inferred from the excavated finds.

### 6.2. Horse phalanges with percussion features and carnivore chewing from Gough's Cave: implications

Our interpretation of a high incidence of carnivore chewing on the horse phalanges matches the results from Parkin *et al*. [127], who recorded a similar prevalence of chew marks on more than a third of the first phalanges. We also note the uniformity in the morphology of the marks that extends to the distribution of the pitting and scoring and the U-shaped profiles of the features, as well as the characteristic fan-like configuration of the scores on distal articular surfaces of the phalanges.

The identification of a high incidence of carnivore tooth marks on cut-marked horse phalanges at Gough's Cave is crucial in the interpretation of human activities undertaken in the cave and their interactions with carnivores at the site. These interactions extend to an analysis of skeletal element representation and the spatial distribution of carnivore-chewed bones in the cave. In their study, Andrews & Fernández-Jalvo [115, p. 61] highlighted differences in the skeletal element representation between the humans and other large mammals at the site. They highlight the ‘extraordinarily high abundance of [horse] phalanges, which are not normally common in human occupation sites’. They also note that, in general, the human remains include a better representation of elements, and that the horse and deer assemblage is marked by relatively abundant cranial elements (especially mandibles), metapodials and phalanges, but a poor representation of the other limb bones. Andrews & Fernández-Jalvo [115] do not provide a detailed explanation for the differences in skeletal element representation between the human and non-human bone assemblages; however, Currant [95, p. 288] presents the problem clearly: ‘Surviving material from the 1927 to 1931 excavations bears all the hallmarks of fairly drastic selection, with a strong bias towards easily identifiable specimens, particularly teeth and foot bones. It is clear from correspondence between Bate and Parry that neither party considered it desirable to retain the large quantity of fragmented bone that had been recovered from Gough's Cave. Their agreement sealed the fate of what would now have been a very valuable taphonomic collection had it survived intact.’ He goes on to contrast this situation with later excavations at the site when ‘Unsorted bone scraps from the 1949, 1950 and 1951 seasons' were retained, which he notes ‘are of considerable value in helping to reconstruct the true nature of the assemblage, even though it is from the inner, less productive parts of the accumulation’ [95, p. 288]. It is also evident that greater care was taken in identifying and selecting the human remains, which includes a substantial component of long-bone shaft fragments and other fragmentary specimens in the collections made prior to 1989.

By taking account of selective post-excavation discard identified by Currant [95], Parkin *et al*. [127] provide a more nuanced interpretation of the taphonomy of the horse and red deer assemblage weaving evidence from their detailed analysis of the butchery evidence with the pattern of carnivore chewing. They note that the body part representation of the horses and red deer varies significantly between different areas of the cave, and that these differences cannot be attributed simply to recovery bias or post-excavation disposal. Parkin *et al*. [127] compared bone assemblages from excavations carried out within the daylight zone inside the cave mouth (1927–1931) with the assemblage from the darker recesses of the cave (1949–1952). Although the assemblage from the cave mouth is depleted in bone flakes (discarded by the excavators and curators), the skeletal element representation of horse and red deer in this area is notable for consisting almost entirely of elements from the heads and limb extremities, with a component of complete bones (tarsals, phalanges, accessory metapodials) and a marked under-representation of upper limb elements. It is notable that all the horse phalanges are from this part of the cave. By contrast, the assemblage from the darker recesses of the cave is characterized by a predominance of shaft fragments from meat- and marrow-bearing upper limb bones and ribs, but with very few fragments of articular ends and no complete bones.

Another key observation noted by the excavators is that lithic artefacts became increasingly rare towards the back of the cave, whereas bones from both areas showed a high incidence of cuts and thus human activity. In addition, Parkin *et al*. [127] noted that a high proportion of horse and red deer bones from both areas showed signs of carnivore gnawing, indicating that the carnivores were present in the cave and scavenged on the bones. Based on these observations, Parkin *et al*. [127, pp. 315–316] propose that carnivores transported the meat- and marrow-bearing bones to the back of the cave from areas nearer the front where they were originally deposited by the human occupants. They suggest that the elements transported to the back of the cave still retained scraps of edible soft tissue, which attracted the attention of carnivores.

**Figure 14.**
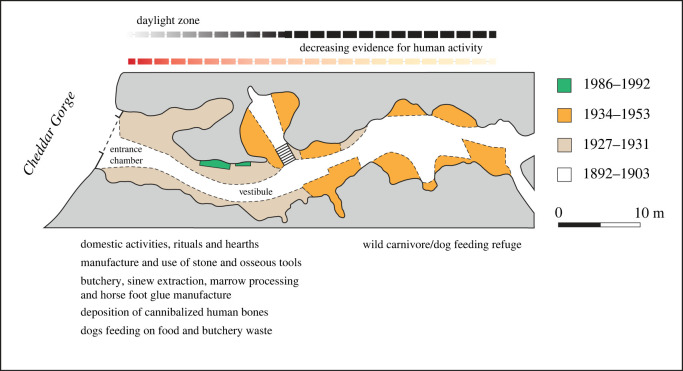
Plan of the Entrance Chamber and Vestibule of Gough's Cave, showing areas investigated by archaeologists and an interpretation of Magdalenian and carnivore activities inferred from the distribution of artefacts and modified bones. Plan based on Jacobi [124] and Stringer [131].

One intriguing question that remains unanswered is why the gnawed first phalanges were not carried by carnivores with the meat- and marrow-bearing bones to the back of the cave, to the areas which Parkin *et al*. [127] identify as carnivore denning places (figure 14). To address this issue, it is crucial to identify the specific types of carnivores present at the site and determine whether they coexisted with humans or arrived at a later time, after the humans had vacated the cave.

Determining the identity of the carnivore(s) responsible for modifying the bones is a challenging task. According to Parkin *et al*. [127], the ‘gnawing was usually little more than scoring of the bones with tooth marks, but destroying little of them’ [127, p. 314]. From our observations on the extent and pattern of the chewing marks, as well as the size of the tooth scores and pits, it appears that medium-sized canids, such as wolves or domestic dogs, were involved in the modification of the bones. Both wolf and domestic dog remains are present in the Gough's Cave Lateglacial assemblage, with the latter being the most common carnivore at the site (Parfitt *et al*., work in progress). It is also possible that other carnivores, such as foxes, which are represented at the site by both red fox (*Vulpes vulpes*) and Arctic fox (*Vulpes lagopus*), lynx (*Lynx lynx*) or brown bears (*Ursus arctos*), may also have fed on the butchery waste after the cave was abandoned by humans.

Based on the available evidence, it is probable that the distribution of bones and carnivore gnawing at Gough's Cave is the result of multiple stages of activity that involved humans (processing large mammals for a range of resources, including sinews and glue extraction), Magdalenian dogs and a later stage when wolves and foxes fed on the scraps after the site was abandoned by humans and their dogs. In this scenario, Magdalenian dogs probably played a role in the initial carnivore modification of the bones and may have selectively picked out the first phalanges and marrow-cracked metapodials from the butchery waste for chewing, as an additional source of nutrients, or as a palliative during periods of boredom.

### 6.3. Origins and development of osseous knapping tools

The origin and development of organic knapping tools are one of the least well-understood topics in Palaeolithic artefact research, and there are significant gaps in the record. However, results from recent analyses of the earliest osseous knapping tools from Boxgrove and some of the youngest Upper Palaeolithic examples from Gough's Cave have shed new light on the importance of these tools in the Palaeolithic.

Gaps in this record are exemplified by the discovery at Boxgrove of a diverse range of 500 000-year-old osseous knapping tools, which include at least three antler flint-knapping hammers. Previously, only modern humans were thought to be capable of making hammers in antler [14]. The use of antler hammers by early Middle Pleistocene hominins represents an important technological advance. It records the selection and working of a novel raw material that combines strength and flexibility, which made antler hammers ideal for detaching thin and long flakes necessary to produce thinner and more symmetrical handaxes. Another important aspect of these knapping tools is the presence of heavy wear on the working end, suggesting they were ‘carefully curated, carried around, and used to make a large number of handaxes' [14].

The antler hammers at Boxgrove were part of a tool kit that included a range of hard (stone) and bone (soft) hammers made from the epiphyses of long bones and the shaft fragments of limb bones from various large mammal species. Some of the bone percussors were also curated, as evidenced by an imported horse acetabulum from the HBS. Another aspect of the bone tool technology at Boxgrove is illustrated at the HBS by bone fragments from marrow processing that were used to remove a few resharpening flakes before being discarded on the spot. The selection and varied use of osseous knapping tools at Boxgrove reflect a more complex and flexible approach to acquiring and using knapping hammers than hitherto anticipated, with important implications for understanding the capabilities of Lower Palaeolithic hominins [34]. At Boxgrove, we see some of the earliest indications of a curated technology, which contrasts with the view that it was not until the Upper Palaeolithic that the use of curated tools becomes a key component in technological behaviour [7,150–152].

The knapping tools from Gough's Cave represent one of the youngest examples of a Palaeolithic knapping tool kit. Our study of the Gough's Cave knapping tools highlights some unexpected findings that challenge previous assumptions about Magdalenian knapping tools. These tool kits are generally believed to have included a greater variety of curated implements, including hammerstones, antler hammers, abraders, antler punches and pressure-flakers that are linked to the range and diversity of tool types made on carefully manufactured blades [153]. The expectation that antler hammers, which are the dominant type of knapping tool known from continental Upper Palaeolithic sites, is not supported by our analysis of the Gough's Cave assemblage in which we identify a diversity of ad hoc and curated organic knapping tools and a complete absence of antler soft hammers. At Gough's Cave, the flint-knappers selected horse metapodials as hammers to detach blades and horse teeth and a phalanx were used for pressure flaking and gentle percussion to shape or rejuvenate tool edges [84]. The use of unmodified bones in this way typifies earlier Lower and Middle Palaeolithic technologies with none of the worked antler hammers and punches that would be anticipated on a site of this age. This reflects the exceptional longevity of a simple tool technology that first appeared in the Lower Palaeolithic. We suspect that ad hoc knapping hammers were likely a more common component of Upper Palaeolithic knapping tool assemblages, which have been overlooked during excavation or not recognized as tools when the bone assemblages were first analysed.

Interpreting the Gough's Cave and Boxgrove assemblages has benefitted from analysing the knapping tools in parallel with the lithic chaîne opératoire and the spatial distribution of finds [35,84,124]. This work was extended to the analysis of carnivore modifications of the bones, which provide additional insights into the site and assemblage formation processes and the interaction of large carnivores with the humans at these sites. This can be seen in the avoidant behaviour of hyaena scavengers at Boxgrove and the presence of domestic dogs at Gough's Cave, both of which fed on butchery waste and marked bones in ways that resemble knapping damage. The pattern of carnivore alteration at Boxgrove suggests hyaenas were responsible for destroying cancellous bones. At Gough's Cave, the pattern of chewing on the horse foot bones suggests that the dogs shared the same space and diet as humans.

Combining information from the knapping tools, lithic chaîne opératoire and carnivore chewing with the spatial distribution of finds provides additional insights into human behaviour at Gough's Cave and Boxgrove. Although this type of analysis is hampered by biases resulting from the excavation and curation history of the Gough's Cave collection, it has nevertheless been possible to identify a varied range of activities undertaken by its Magdalenian occupants, and to identify carnivore denning areas in darker places towards the rear of the cave. These areas were likely used by canids (domestic dogs, wolves or foxes), who fed on butchery waste carried there from the mouth of the cave. The greater precision for identifying the spatial distribution of hominin activities is possible at the HBS at Boxgrove. At this locality, three-dimensional point recording of every bone fragment and flint object at the HBS at Boxgrove makes it possible to identify links between specific flint-knapping tasks and individual knapping tools. This is complemented by the refitting of bone fragments, which provides additional information on the ad hoc component of the knapping tool assemblage, as well as the movements and interactions of knappers and butchers working around the horse carcass. The clarity of this record is probably unique for an early Middle Pleistocene Acheulean butchery site [35].

**Figure 15.**
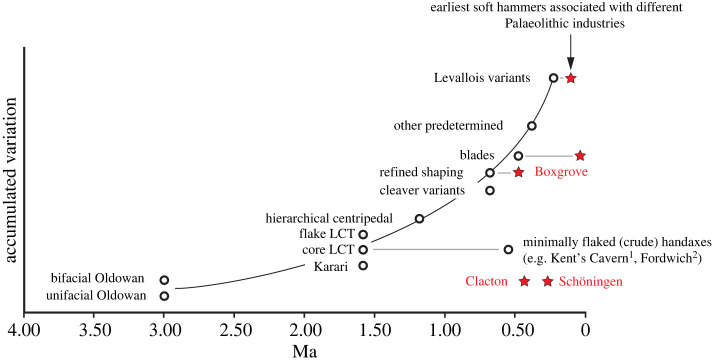
Accumulation of innovations in Palaeolithic technology (after Stout [154]) plotted against the earliest records of soft organic hammers associated with lithic technologies where soft hammer use has been identified from waste flake morphology. Note the transition in Britain of irregular handaxes (large cutting tool (LCT): Kent's Cavern^1^ [155]; Fordwich^2^ [156]) made with hard hammers to later refined shaping in handaxe manufacture involving platform preparation and soft hammers [34]. The late occurrences of simple lithic technologies in western Europe may indicate alternating regional occupations by two or more human lineages with different tool traditions [157].

The paper by Stout [154] opens the discussion to wider issues relating to the archaeological record of osseous knapping tools and their importance for identifying enhanced planning abilities and cognitive complexity. In that paper, Stout established a systematic method for describing the increasing complexity and diversity of early lithic technologies which is summarized in a plot that shows the rates of Palaeolithic culture change inferred from the appearance of new lithic technologies and manufacturing methods. Stout's diagram [154, fig. 2] is modified in figure 15 to incorporate the first evidence for osseous knapping tools associated with the main technological variants.

Stout points out that although the Middle Palaeolithic/MSA is widely ‘considered to represent an order-of-magnitude increase in technological complexity’ with the use of a greater variety of raw material combined to make composite tools, he considers the adoption of specialized knapping tools (such as the Boxgrove antler hammers) in the Late Acheulean as reflecting aspects of a similar level of cognitive fluidity and enhanced planning abilities [154, pp. 586–587]. Elaborately shaped handaxes conforming to this type first appear in Europe at about 0.5 Ma, with Boxgrove handaxes serving as examples of the early emergence of these new types in western Europe. This technology has its origin in Africa, where a transition to ‘smaller, thinner, more regular and symmetrical LCTs [large cutting tools] thought to require the use of a “soft hammer” technique during production’ [154, p. 1054] has its earliest appearance at Isenya, Kenya [158] and Konso, Ethiopia [159] dating to approximately 0.85 Ma. Currently, no unequivocal organic knapping tools are known from African Late Acheulean sites.

The hominins responsible for making many pre-Upper Palaeolithic industries are not reliably established [160], and each lineage may have its own unique trends and trajectories in developing technologies, and these may vary for different components of their toolkit. An example of such a ‘dissociation’ may be seen in the Clactonian and at Schöningen, with technologically simple lithics with the continuation of flake-and-core industries into the late Middle Pleistocene in western Europe. At both sites, bone-knapping tools are a component of the tool technology, as are specialized spears, lances and throwing sticks. Moreover, usewear analyses of the Schöningen lithic tools [44] suggest that some of the pieces may have been hafted, possibly as composite tools incorporating the enigmatic forked sticks (Klemmschafte) as hafts. These elements combine to indicate a high level of cognitive ability, which is not immediately apparent from a technological analysis of the lithic industries in isolation.

Although stone tools provide one indication of technological capabilities in Palaeolithic hominins, integration of this record with the organic technologies and hunting and butchering skills need to be further investigated to establish the extent of differences in ability, manual dexterity and manufacturing skills of different hominin taxa. Such studies may help to identify whether interactions between different groups resulted in cultural exchange that might have led to the transfer of technical skills between groups.

## 7. Conclusion

It is hoped that the various ideas presented here will stimulate discussion and further research, specifically in the area of museum collections, where previous studies may have overlooked osseous knapping tools, warranting their re-evaluation. Unfortunately, specialists studying knapping tools tend to view their findings in isolation from those of other specialists working on the same site, and as a result, the outcomes from these independent lines of evidence have yet to be fully integrated at a comprehensive level. It is only through the meticulous integration of details that new patterns and explanations emerge, as exemplified by the identification of previously unidentified knapping tools and the close relationship between humans and domesticated dogs at the Upper Palaeolithic site of Gough's Cave and the examination of nuances in the use by Acheulean hominins of osseous knapping hammers and their curation and transport from site to site in the wider landscape of Boxgrove.

At a more focused level, the current study has drawn attention to the challenges involved in recognizing and interpreting Palaeolithic osseous knapping tools, which are important markers of early human technological and cultural evolution. The study has highlighted examples of missed and misidentified knapping tools, demonstrating the difficulty of identifying minimally modified bone tools and accurately interpreting surface marks and other modifications in bone assemblages from Palaeolithic sites. The case studies also demonstrate a reality of taphonomic analysis where the imperative is to categorize surface marks and other bone modifications and interpret them based on the causal processes that could have produced them. This requires a detailed understanding of the natural and cultural processes that affect the preservation of bone materials, as well as a sound knowledge of the range of modifications that can be produced by human and non-human agents. The bones examined in this study often exhibit a range of scratches, pits, polishes, grooves and breaks that may have occurred at any stage from the death of the animal to the time of study in the museum collection, making it challenging to assign each feature to a specific human or natural mechanical–chemical agency.

Future research using new imaging methods and reference samples generated from taphonomic experiments will help to refine our understanding of the activities that occurred at these archaeological sites. Despite the challenges of identifying and analysing knapping tools, extending the search for osseous knapping tools in other geographical regions and time periods where osseous knapping tools remain elusive is essential to gain a deeper understanding of the technological and cultural evolution of early humans. Although the task is challenging, the insights gained from the study of knapping tools will provide invaluable information about the cognitive, technological and social development of early humans.

## Data Availability

Permission to study the specimens was granted by curators of fossil mammals at the Natural History Museum, London, where the collections are housed. The specimens are accessible through a request to the curator in charge of the Palaeontology (fossil mammal) collections. Research materials, including images of the specimens and a dataset of the Gough's Cave horse phalanges (NHM collection) with a summary of taphonomic modifications observed by the authors, are available in the electronic supplementary material [161].
